# A user-friendly, high-throughput tool for the precise fluorescent quantification of deoxyribonucleoside triphosphates from biological samples

**DOI:** 10.1093/nar/gkaa116

**Published:** 2020-02-27

**Authors:** Judit Eszter Szabó, Éva Viola Surányi, Bence Sándor Mébold, Tamás Trombitás, Mihály Cserepes, Judit Tóth

**Affiliations:** 1 Institute of Enzymology, Research Centre for Natural Sciences, Budapest 1117, Hungary; 2 Department of Applied Biotechnology and Food Sciences, Budapest University of Technology and Economics, Budapest 1111, Hungary; 3 Department of Experimental Pharmacology, National Institute of Oncology, Budapest, Hungary

## Abstract

Cells maintain a fine-tuned, dynamic concentration balance in the pool of deoxyribonucleoside 5′-triphosphates (dNTPs). This balance is essential for physiological processes including cell cycle control or antiviral defense. Its perturbation results in increased mutation frequencies, replication arrest and may promote cancer development. An easily accessible and relatively high-throughput method would greatly accelerate the exploration of the diversified consequences of dNTP imbalances. The dNTP incorporation based, fluorescent TaqMan-like assay published by Wilson *et al.* has the aforementioned advantages over mass spectrometry, radioactive or chromatography based dNTP quantification methods. Nevertheless, the assay failed to produce reliable data in several biological samples. Therefore, we applied enzyme kinetics analysis on the fluorescent dNTP incorporation curves and found that the Taq polymerase exhibits a dNTP independent exonuclease activity that decouples signal generation from dNTP incorporation. Furthermore, we found that both polymerization and exonuclease activities are unpredictably inhibited by the sample matrix. To resolve these issues, we established a kinetics based data analysis method which identifies the signal generated by dNTP incorporation. We automated the analysis process in the nucleoTIDY software which enables even the inexperienced user to calculate the final and accurate dNTP amounts in a 96-well-plate setup within minutes.

## INTRODUCTION

Despite the longstanding need and desire for the precise quantification of cellular levels of deoxyribonucleoside triphosphates (dNTP), none of the existing methods could become routine molecular biology procedure. Besides the general importance of dNTP homeostasis, recent studies have started to unveil its key roles in oncogenic progression (1–7), antiviral defense (8), aging (9–11), cell cycle control (3,4,12,13) and antibody hypermutation (14). To understand the role of dNTP concentration changes in these processes, the most valuable information could be obtained by measuring intracellular dNTP levels directly in the cell. As no method is available to do so, we would need to reliably evaluate the dNTP concentration of cellular lysates, at least. For this, several high performance liquid chromatography (HPLC) based methods and enzymatic assays have been developed. Each method has its advantages and disadvantages but in general, the biggest challenge of dNTP quantitation is that other nucleotides, primarily ribonucleoside triphosphates (rNTPs) are present in much higher concentration in the cell lysate than dNTPs and the separation of the various nucleotides from each other and from the sample matrix is not straightforward.

For HPLC analysis, rNTPs may be removed by boronate chromatograpy (15,16) (compatible only with UV detection (17)) or by specific degradation using chemical or enzymatic methods (17). But even at a 99% efficient removal of rNTP-s, the remaining 1% can interfere with dNTP quantitation (17). HPLC may be coupled with either UV or mass spectrometry (MS) detection. The main advantage of the UV-based HPLC methods is that they can also be used to measure the rNTPs which provides a good control to know whether nucleotide extraction worked properly. UV detection has the disadvantage of the requirement of a large sample size.

The HPLC coupled MS detection provides the highest sensitivity, but it has several disadvantages. Generally, the sample extraction and chromatographic separation processes are time-consuming and these methods require specialized labs and high level of expertise in analytics. The optimal separation of nucleotides is commonly achieved using ion paring agents (e.g. the most often used diethylamine). However, these chemicals are incompatible with other standard uses of the MS instrument due to their strong retention inside the mass spectrometer (18). Therefore, the reported HPLC-MS based methods cannot simply be applied on any instrumentation. The mobile phase of the HPLC method, largely responsible for the separation of nucleotides, is needed to be tailored to make it compatible with the available instrumentation and with its other uses. Furthermore, isobaric nucleotide pairs sharing the same mass cannot be distinguished using MS ((deoxy)cytidine vs. (deoxy)uridine isobars (19), deoxyguanosine versus adenosine isobars (17)). A recently published method surmounted the latter difficulties by using an ion-pair-free mobile phase and it is able to detect the eight canonical dNTPs and rNTPs in the biological sample with high sensitivity (20).

Enzyme based methods include the DNA polymerization based radioisotope incorporation technique (21,22), the single nucleotide incorporation based radioisotope technique (23), the TaqMan assay-like fluorescence method by Wilson *et al.* (24), and the most recent DNA polymerase/restriction enzyme based isothermal DNA machine (not yet tested by the scientific community according to the literature) (25). Amongst the enzymatic methods, the most widely used is the DNA polymerization based radioisotope incorporation method. Although this technique is sensitive and reliable, it is labour-intensive and hardly applicable as a high throughput method. This also applies for the single nucleotide incorporation method (23). DNA polymerization based methods may also interfere with the presence of high concentration of rNTPs (22) which can be overcome by the appropriate choice of DNA polymerase and its concentration. These conditions were already considered in the TaqMan assay-like fluorescence method, which relies on similar principles as the polymerization based radioisotope incorporation method using a different detection technique (24). This fluorescence method appeared to revolutionize dNTP concentration measurements with its basic molecular biology requirements and high throughput manner (24).

As we have been interested in dNTP metabolism, we set out to adapt a method for dNTP quantitation for everyday use. Although we applied the radioactive assay successfully before (26), we wished to analyze a high number of samples in a more high throughput manner. We initiated HPLC-MS trials in collaboration, which failed due to the aforementioned incompatibility of the ion pairing organic solvents with other uses of the MS instrument. The fluorescence method by Wilson *et al.* (24) seemed the only option for high-throughput, quantitative dNTP measurements. However, the assay failed in several biological samples, the data analysis method described in the paper yielded negative values. It was particularly difficult to measure dATP. We analyzed the 29 publications citing this paper so far to find out if fellow researchers could apply this method with success. Of the 14 papers that actually applied the dNTP quantification method, detailed method description and exact dNTP quantities are presented in only 3, including a paper from the same authors (27–29). In the other citing publications, the results are presented as relative dNTP levels or extremely raw data (fluorescence intensity) which do not allow the evaluation of the applicability of this method. Matsuura *et al.* mentions that they modified the original method by Wilson *et al.* to ‘reduce the background noise caused by polymerase-mediated hydrolysis of the probe’ (30). Unfortunately, the modified method was not included in the paper and is still not published. Our own experience and the citing literature indicated that this promising method needed further elaboration in order to become an easily accessible and popular means of dNTP quantification.

Here, we report that the major artefacts hindering the applicability of the Wilson *et al.* method originate from the polymerization independent 5′-3′ exonuclease activity of Taq polymerase and from kinetic inhibition by the biological sample. Both of these disturbing effects can be overcome by our altered assay conditions and by a novel data analysis method. We apply a kinetic treatment of the assay curves which enables the separation of the polymerization dependent and independent processes. We also developed a software offering streamlined data analysis. Using the nucleoTIDY software, the whole analysis process including the kinetic treatment of the assay curves, the construction of the calibration curves and the calculation of the dNTP quantity in the actual biological samples takes just a few minutes for a 96-well-plate. These developments make dNTP quantification widely available to the scientific community in any biological samples.

## MATERIALS AND METHODS

### Strains and growth conditions

The *Mycobacterium smegmatis* mc^2^155 strain used for the experiments was grown in Lemco liquid culture or on solid Lemco plates with the addition of 15 g l^−1^ Bacto agar as described previously (31). MES-SA human uterine sarcoma cell line was obtained from ATCC. The cells were cultured in DMEM (Gibco) supplemented with 10% fetal bovine serum (Gibco), 5 mM l-glutamine (Gibco), and 50 U/ml penicillin/streptomycin solution (Life Technologies). Cell culture flasks were grown up to 90% confluency before harvesting in order to examine monolayer cells.

### dNTP pool extraction and sample handling

#### Bacterial cells

Cells were grown until the culture reached the mid-exponential phase OD_600_ = 0.7. The total CFUs were determined for each culture. The cultures were centrifuged (20 min, 3600 g, 4°C) and the cell pellets were extracted in precooled 0.5 ml 60% methanol overnight at −20°C. After 5 minutes boiling at 95°C, cell debris were removed by centrifugation (20 min, 16 000 g, 4°C). The methanolic supernatant containing the soluble dNTP fraction was vacuum-dried (Eppendorf) at 45°C. Extracted dNTPs were dissolved in 50 μl nuclease-free water and stored frozen until use.

#### Human cells

Cultured cells were rinsed with phosphate-buffered saline (PBS) to remove residual media. dNTPs were extracted based on Wilson *et al.* (24) with a few modifications. Briefly, adherent cells were detached by trypsin, resuspended gently in 10 ml of ice-cold PBS and 500 μl aliquot was removed for cell number determination using a hemocytometer. The samples were centrifuged for 10 min at 3000 g at 4°C, cell pellets were resuspended in 500 μl of ice-cold 60% methanol, then placed at -20°C overnight. The sample was afterwards boiled at 95°C for 3 min, then centrifuged (16 000 g for 5 min at 4°C). The supernatant was transferred into a new tube and evaporated under centrifugal vacuum at 45°C. The resultant pellet was resuspended in 50 μl nuclease-free water ready to assay or stored frozen until use.

#### Handling of the dNTP extracts

dNTP samples, if not properly handled, are prone to degradation. To avoid uncontrolled loss of dNTP within the sample, the extracts should be analysed as soon as they are ready or else should be stored in a deep freezer considering that storage in too small aliquots leads to significant concentration of the samples. Freezing must be done in liquid nitrogen. Repeated freeze-thaw cycles should be avoided and all analyses are best completed in a reasonably short time. The pH of the sample and reaction mix should be well controlled.

### Reaction conditions, data acquisition and evaluation

**Figure 1.**
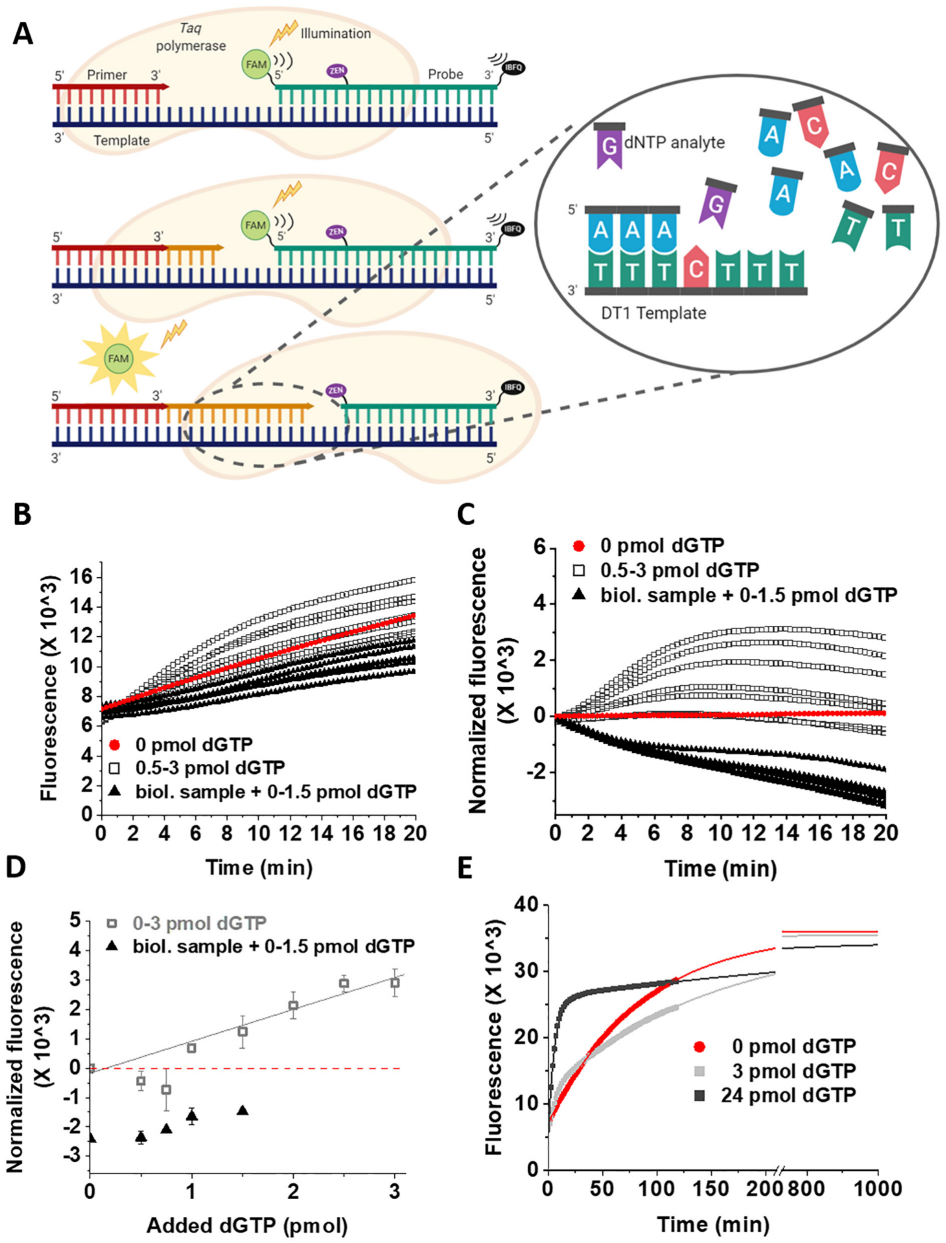
Measurement of cellular dGTP concentration with dT1 template in human sample using the method by Wilson *et al.* (24) (**A**) The schematic representation of the assay. The yellow star symbolizes the release of FAM (light green circle) from the probe (dark green DNA) previously quenched by the ZEN (purple oval) and IBFQ (black oval) quenchers. The light orange shape symbolizes the *Taq* polymerase, while DNA strands are symbolized by comb shapes as follows: dark blue, template; red, primer; orange, newly synthesized DNA; dark green, probe. (**B**) Raw reaction curves in the absence of biological sample (0–3 pmol dGTP /calibration reaction) and in the presence of human sample (0–1.5 pmol dGTP standard addition + dNTP extract from 5 × 10^5^ cells /reaction). Note, that the blank reaction (0 pmol dGTP) also produces signal, which complication was eliminated by Wilson *et al.* via subtracting blank curves from sample curves (shown in panel C). (**C**) Reaction curves normalized according to Wilson *et al.* (**D**) Calibration curve (0–3 pmol dGTP) in the absence of biological sample, and standard addition points in the presence of biological sample derived from reading the fluorescence of panel C at 15 min. Continuous lines are linear fits to the data yielding the following parameters: calibration without biological sample: intercept = −115±145, slope = 1056 ± 113, *R*^2^ = 0.98. In case of the calibration points, the negative data points were omitted from the fitting. Data and errors represent the average and standard deviation of technical parallels (*n* = 2). (**E**) Fitting the raw calibration curves (scatter plots) with exponential function (continuous line). In case of the blank reaction (0 pmol dGTP) single exponential equation (Equation 1) could be fitted to the raw reaction curve, while in the presence of the specific dNTP (here dGTP), the reaction could be well described with a double exponential (Equation 2). The parameters yielded from the exponential fits were as follows: 0 pmol dGTP: A = −28 869, *k*_obs_ = 2.0 × 10^−4^ s^−1^, *y*_0_ = 35 978; 3 pmol dGTP: *A*_1_ = −6393, *k*_1obs_ = 2.5 × 10^−3^ s^−1^, *A*_2_ = −23 485, *k*_2obs_ = 1.1 × 10^−4^ s^−1^, *y*_0_ = 35540; 10 pmol dGTP: *A*_1_ = −8480, *k*_1obs_ = 2.9 × 10^−3^ s^−1^, *A*_2_ = −20 879, *k*_2obs_ = 5.0 × 10^− 5^ s^−1^, *y*_0_ = 34 454.

**Figure 2.**
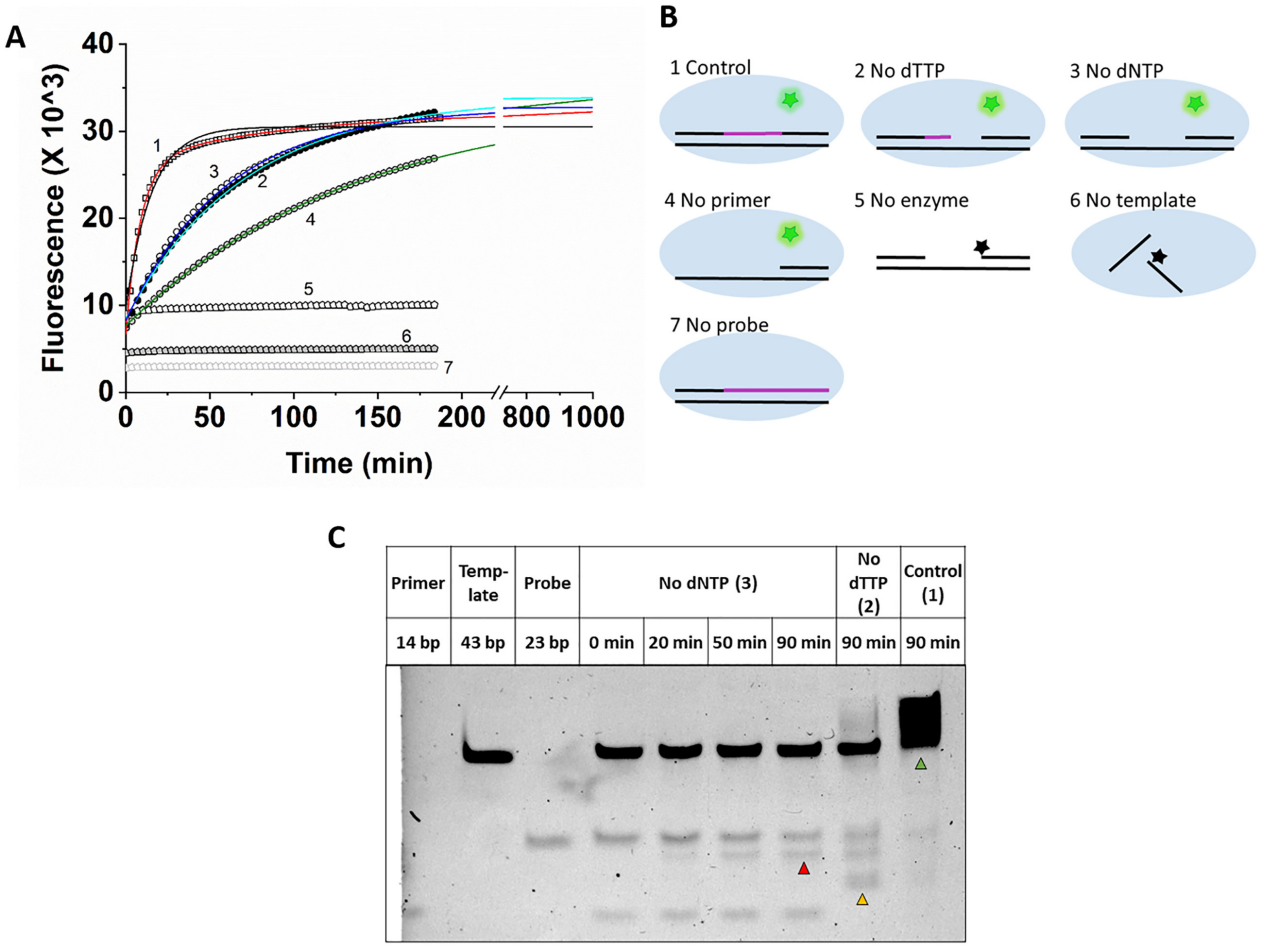
Assay background originates from dNTP incorporation independent 5′-3′ exonuclease activity of AmpliTaq Gold™ polymerase. (**A**) An analysis of the dTTP assay was chosen using the dT1 template. The data presented in Panel A represent the reaction curves obtained under the conditions schematically shown in panel B. Continuous lines represent the exponential fits to the data. Parameters are as follows: 10 pmol dTTP (1), red: A_1_ = −19 786, *k*_1obs_ = 1.91 × 10^−3^ s^−1^, *A*_2_ = −5787, *k*_2obs_ = 1.79 × 10^−4^ s^−1^*y*_0_ = 32 213; 0 pmol dTTP (2), cyan: *A*_1_ = −25 382, *k*_obs_ = 2.37 × 10^−4^ s^−1^, *y*_0_ = 33 832; No aspecific dNTP added (3), blue: *A*_1_ = −24 458, *k*_obs_ = 2.79 × 10^−4^ s^−1^, *y*_0_ = 32 718; No primer added (4), green: *A* = −26 023, *k*_obs_ = 1.22 × 10^−4^ s^−1^, *y*_0_ = 33 659. A single exponential function fitted to the ‘10 pmol dTTP’ (1) data is shown as continuous black line. (**B**) Panel B schematically explains the constitution of the different assays. The incorporated dNTP-s are colored magenta. (**C**) Denaturing urea-PAGE gel demonstrating the dNTP incorporation independent 5′-3′ exonuclease activity of Taq polymerase. Numbers in parenthesis correspond to those in panels A and B. Red arrow: hydrolyzed probe; yellow arrow: elongated primer; green arrow: duplicated template.

**Figure 3.**
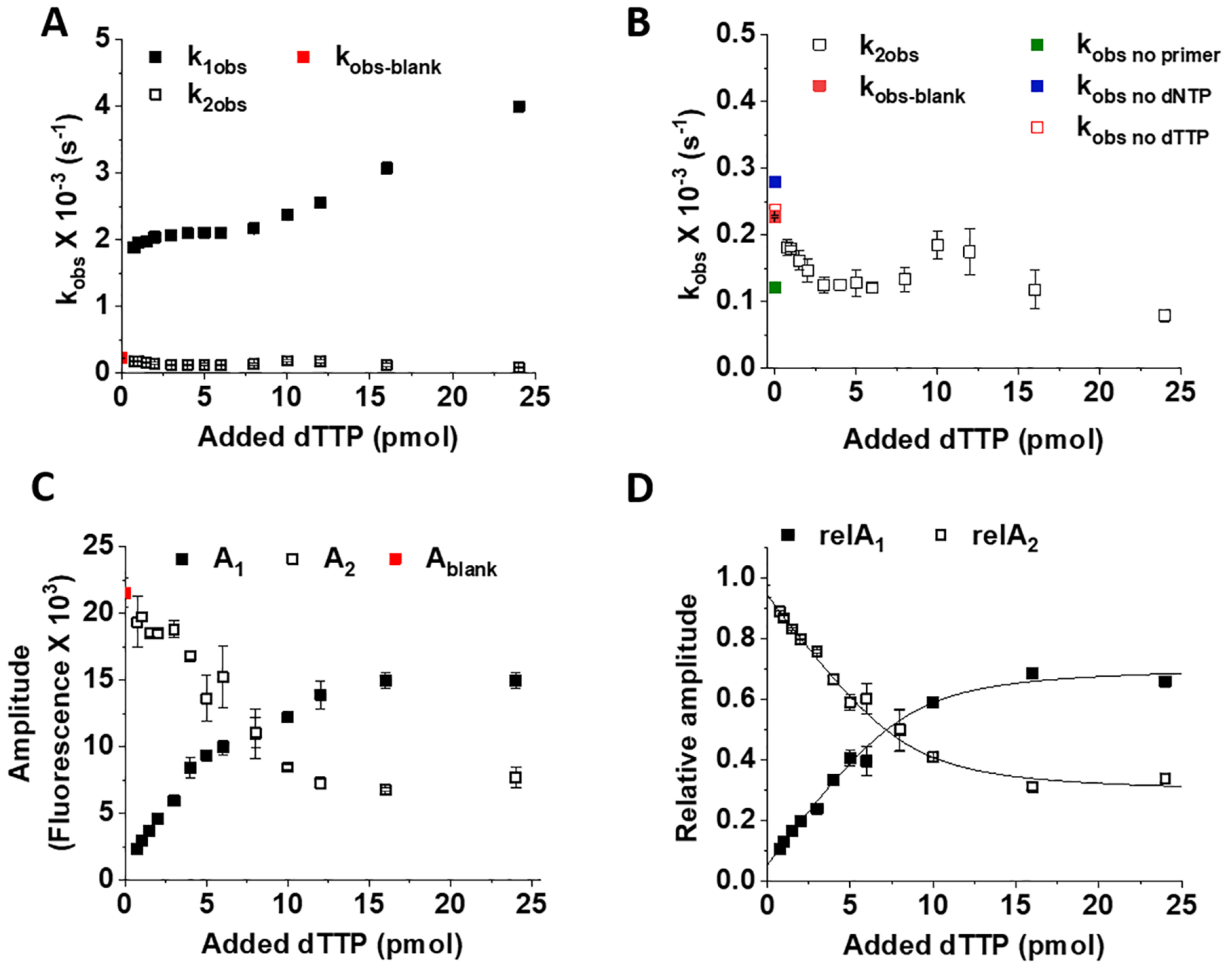
Background exonuclease activity of AmpliTaq Gold™ polymerase competes with specific dNTP incorporation in a concentration dependent manner. Concentration is understood to be pmol dTTP in constant reaction volume. (**A**) Concentration dependence of the observed rate constants of the two kinetic phases in the assay (*k*_1obs_ and *k*_2obs_, respectively). (**B**) Concentration dependence of the observed rate constant of the slower phase (*k*_2obs_). The observed rate constants of the background reaction presented in Figure 2A are also shown as *k*_obs no primer_, *k*_obs no dNTP_, *k*_obs no dTTP_ (blank). (**C**) Concentration dependence of the amplitudes of the two phases (A1 and A2, respectively). (**D**) Concentration dependence of the relative amplitudes of the two phases (relA_1_ and relA_2_, respectively). The amplitudes were normalized compared to the total amplitude of the reaction (A1 + A2). The relative amplitudes could be fitted with quadratic binding equation (Equation 3) yielding the following parameters: *A*_quad_ = 0.660 ± 0.036, *S* = 0.054 ± 0.002, *c* = 8.345 ± 0.453, *K*_app_ = 0.741 ± 0.290 for relA_1_, and *A*_quad_ = −0.660 ± 0.036, *S* = 0.946 ± 0.002, *c* = 8.345±0.453, *K*_app_ = 0.741 ± 0.290 for relA_2_. Data represent the average and SD of two technical parallels for each panel.

**Figure 4.**
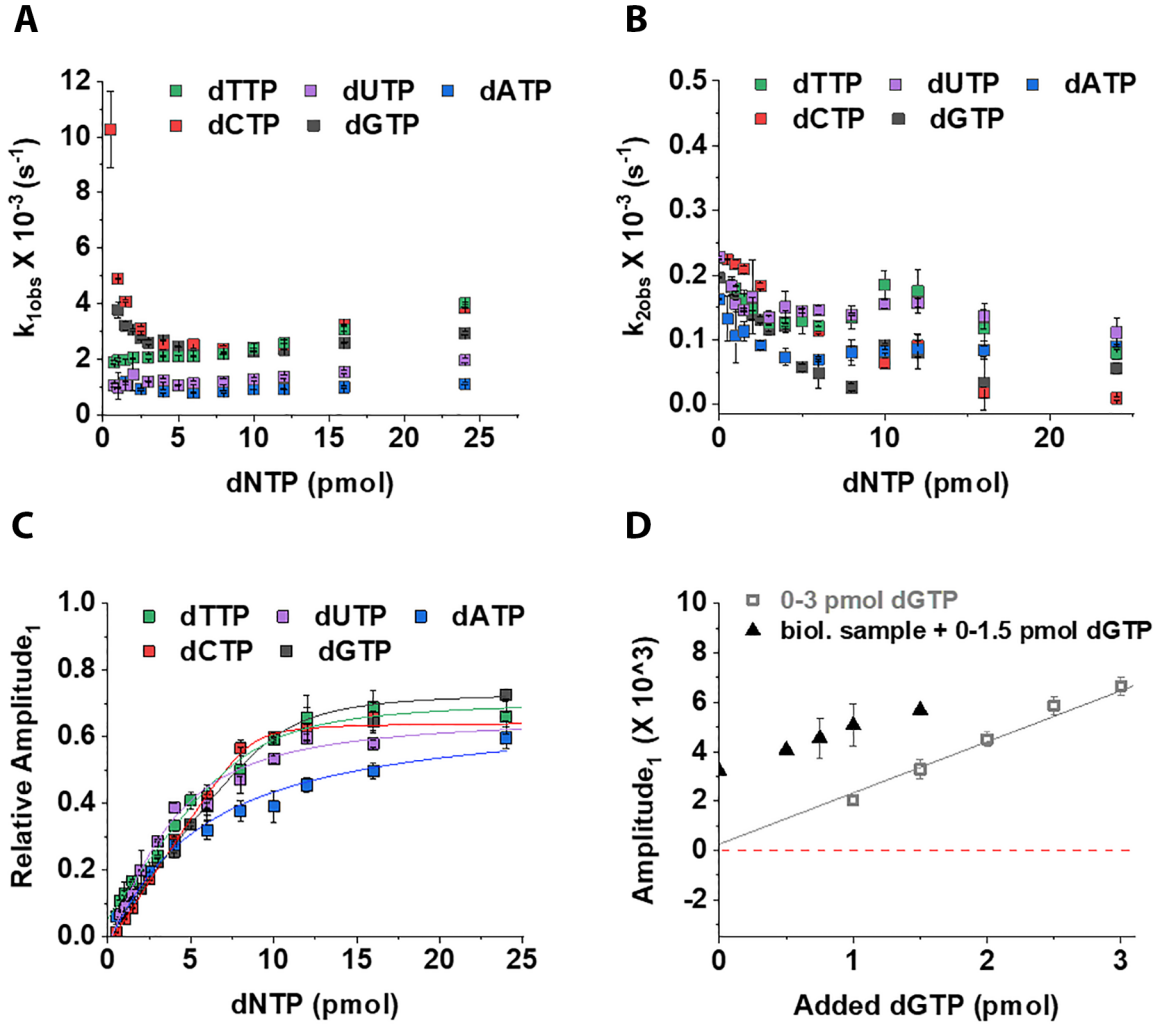
Separation of the reaction phases for dNTP quantitation. (**A**) For each dNTP-s, the dependence on the amount of added specific dNTP is shown for (A) the observed rate constants of the fast, dNTP incorporation associated phase (*k*_1obs_); (**B**) the slow, aspecific phase (*k*_2obs_); (**C**) the relative amplitudes of the fluorescence change of the fast, dNTP incorporation associated phase. Relative amplitudes could be fitted with the quadratic binding equation (Equation 3) yielding the following parameters: *A*_quad_ = 0.70 ± 0.10, *S* = 0.03 ± 0.03, *c* = 3.60 ± 2.10, *K*_app_ = 1.67 ± 1.48 for dUTP; *A*_quad_ = 0.59 ± 0.11, *S* = −0.01±0.03, *c* = 3.94 ± 3.09, *K*_app_ = 2.29 ± 2.12 for dATP, *A*_quad_ = 0.77 ± 0.10, *S* = −0.03 ± 0.00, *c* = 8.57±0.31, *K*_app_ = 0.10 ± 0.13 for dCTP and *A*_quad_ = 0.73 ± 0.06, *S* = 0.01 ± 0.00, *c* = 10.41±0.72, *K*_app_ = 0.39 ± 0.26 for dGTP. For dTTP the same curve is presented in panels A–C as in Figure 3. (**D**) Comparison of calibration curves in the presence and absence of biological sample using the A1 parameter. The data points were derived from the measurement presented in Figure 1. The continuous line is a linear fit to the data (*R*^2^ = 0.98). Note that using the A_1_ parameter, dGTP could be quantified in the biological sample in contrast to the analysis method presented in Figure 1D. Also, the added extra dGTP could be recovered. We calculated the recovery for the presented standard addition points to be 85 ± 4% (mean + SD, note that this measurement was still conducted under the original, suboptimal conditions). Also note that the first three data points of the calibration curve are not presented here, since the contribution of the fast, specific phase to the total amplitude was too small for reliable fitting. Data represent the average and SD of two technical parallels for each panel.

We applied the reaction conditions described in Wilson *et al.* (24) to obtain the results shown in Figures 1–4 and Supplementary Figures S1–S4. Briefly, 10 pmol template, 10 pmol probe and 10 pmol NDP1 primer was present per 25 μl reaction. The concentration of each non-specific dNTP was kept at 100 μM. The AmpliTaq Gold™ DNA Polymerase (Applied Biosystems™, Thermo Fisher Scientific) was used at 0.825 unit/reaction while the VWR^®^ TEMPase Hot Start DNA Polymerase (VWR) was used at 0.9 unit / reaction in the presence of 2.5 mM MgCl_2_. To record calibration curves, the reaction was supplied with 0–10 pmol or 0–20 pmol specific dNTP depending on the applied dT1 or dT2 template, respectively. The sensitivity of the dNTP quantitation reaction is highly determined by the number of specific dNTPs to be incorporated. The dT1 template allows the incorporation of only one molecule of specific dNTP (Figure 1A), and is applicable in the 0–10 pmol dNTP range, while the dT2 template allows the incorporation of two specific dNTPs, and is applicable up to 20 pmol dNTP with a lower sensitivity. The sequences of the applied primers and probes are presented in Table 2 (24). Fluorescence was recorded every 17 seconds in a CFX96 Touch™ Real-Time PCR Detection System for 25 min, or longer. We used FrameStar^®^ 96-Well Skirted PCR Plates with black wire and white wells and sealed with Eppendorf adhesive PCR films. In a few measurements, the QuantStudio 5, QuantStudio 1 (Thermo Fisher), and the AriaMx (Agilent) instruments were also used with plates recommended by the manufacturers. The choice of instrument did not seem to affect the results. DNA primers were from Sigma (standard purification), while the fluorescence probes were ordered from Integrated DNA Technologies subjected to HPLC purification. The concentration of each stock of the dNTPs and primers were determined by measuring the absorbance at 260 nm. The VWR^®^ TEMPase Hot Start DNA Polymerase catalysed reaction proved to be faster than the AmpliTaq Gold™ polymerase catalyzed reaction, therefore, data were collected for shorter time, and fluorescence was read out earlier than with the AmpliTaq Gold™ polymerase (cf. Supplementary Figure S1). The thermal profile for the AmpliTaq Gold™ polymerase: 95°C 10 minutes, 60°C for varying times (cf. Table 1); and for the VWR^®^ TEMPase Hot Start DNA Polymerase: 95°C 15 min, 60°C 13 s × 260 cycle for dATP measurement. For dCTP, dGTP and dTTP measurements, the polymerization temperature needed to be decreased to 55°C for the VWR^®^ TEMPase Hot Start DNA Polymerase to be able to record the initial fluorescence change. Later, to optimize the reaction conditions for amplitude based dNTP quantification, we varied the template, probe, primer, MgCl_2_ and polymerase concentrations (Cf. Results, Table 1 and Supplementary Table SI).

**Table 1.** Analysis of assay performance using dT1 templates and AmpliTaq Gold™ polymerase or VWR® TEMPase Hot Start DNA Polymerase

												Recovery		
	Assay conditions	qPCR	Data acquisition	*T* (°C)	Calibration range (pmol)	*R* ^2^	LOD^a^ (pmol)	LOQ^a^ (pmol)	Accuracy low, high (%)	Interassay CV (%)	Intraassay CV (%)	(%)	CFU or cell^b^ /	Sample type
**dTTP**	20 pmol TPP, 0.875 u AmpliTaq Gold™, 5 mM MgCl_2_	BioRad CFX96	14 s for 600 cycles	60	0.63–12	0.986 ± 0.006	0.41 ± 0.07	0.68 ± 0.12	100 ± 14 100 ± 6	9.6 ± 7.0	7.8 ± 5.3	105	1 × 10^7^	*M. smegmatis*
												100	1 × 10^8^	*S. aureus*
												98	2 × 10^5^	MES-SA
	10 pmol TPP 0.9 unit VWR® TEMPase, 2 mM MgCl_2_	QuantStudio 1	13 s for 260 cycles	55	0.1–6	0.999	0.05	0.10	102 ± 7 109 ± 4	5.5 ± 6.5	5.3 ± 3.7	101	1 × 10^5^	MES-SA
**dCTP**	10 pmol TPP, 0.438 u AmpliTaq Gold™, 2.5 mM MgCl_2_	BioRad CFX96	13 s for 60 s then 80 s for 128 cycles	60	1–8	0.991 ± 0.001	0.70 ± 0.13	1.10 ± 0.03	98 ± 4 100 ± 5	7.4 ± 4.3	6.9 ± 4.6	98	5 × 10^6^	*M. smegmatis*
												76	1 × 10^7^	*M. smegmatis*
												92	1 × 10^8^	*S. aureus*
												94	1 × 10^6^	MES-SA
	10 pmol TPP, 0.9 u VWR^®^ TEMPase, 2 mM MgCl_2_	QuantStudio 1	13 s for 260 cycles	55	0.6–10	0.992	0.30	0.50	101 ± 3 100 ± 9	6.5 ± 4.4	5.2 ± 2.9	92	6 × 10^7^	*M. smegmatis*
**dGTP**	10 pmol TPP, 0.438 u AmpliTaq Gold™, 2.5 mM MgCl_2_	BioRad CFX96	13 s for 60, then 80 s for 128 cycles	60	0.5–10	0.992 ± 0.001	0.50 ± 0.07	0.83 ± 0.11	99 ± 21 99 ± 4	12.6 ± 10.2	9.4 ± 5.7	102	1 × 10^7^	*M. smegmatis*
												109	5 × 10^5^	MES-SA
	10 pmol TPP, 0.438 u AmpliTaq Gold™, 5 mM MgCl_2_	QuantStudio 1	13 s for 100, then 80 s, for 200cycles	57	0.25–8	0.997	0.18	0.33	99 ± 15 98 ± 6	ND	ND	115	5 × 10^7^	*E. coli*
												91	4 × 10^7^	*S. aureus*
	10 pmol TPP, 0.9 u VWR^®^ TEMPase, 3.5 mM MgCl_2_	QuantStudio 1	13 s for 260 cycles	55	0.4–10	0.999	0.11	0.19	99 ± 2 101 ± 5	5.6 ± 4.9	4.5 ± 2.5	101	6 × 10^6^	*M. smegmatis*
**dATP**	20 pmol TPP, 0.875 u AmpliTaq Gold™, 5 mM MgCl_2_	BioRad CFX96	17 s for 700 cycles	60	1–8	0.991 ± 0.004	0.59 ± 0.26	1.06 ± 0.26	95 ± 7 100 ± 0.1	12.0 ± 0.1	7.9 ± 1.6	92	1 × 10^6^	MES-SA
												73	1 × 10^8^	*S. aureus*
												102	1 × 10^7^	*M. smegmatis*
	15 pmol TPP, 0.9 u VWR^®^ TEMPase, 5 mM MgCl_2_	BioRad CFX96	13 s for 260 cycles	60	1.5–15	0.977	1.68	2.81	104 ± 3 99 ± 1	10.1 ± 2.4	3.3 ± 1.8	97	1 × 10^8^	*M. smegmatis*

^a^LOD and LOQ were calculated by the two methods presented in the methods sections. If the mathematical calculation of LOD and LOQ gave lower value than the lowest resolvable calibration point, then the lowest calibration point should be considered as the LOD.

^b^dNTP extract from the given number of CFU (for bacteria) or cells (for eukaryotic cells) / well.

TPP: template, probe, primer (NDP1), u: unit.

**Table 2.** Sequences of oligonucleotides used in the assays

Name	Sequence (5′→3′)
NDP-1 primer	CCGCCTCCACCGCC
FAM-dTTP probe	**6-FAM/** AGGACCGAG **/ZEN/** GCAAGAGCGAGCGA **/IBFQ**
FAM-dATP probe	**6-FAM/** TGGTCCGTG **/ZEN/** GCTTGTGCGTGCGT **/IBFQ**
FAM-dGTP probe	**6-FAM/** ACCATTCAC **/ZEN/** CTCACACTCACTCC **/IBFQ**
FAM-dCTP probe	**6-FAM/** AGGATTGAG **/ZEN/** GTAAGAGTGAGTGG **/IBFQ**
dTTP-DT1 template	TCGCTCGCTCTTGCCTCGGTCCTTT **A** TTTGGCGGTGGAGGCGG
dTTP-DT2 template	TCGCTCGCTCTTGCCTCGGTCCTTT **A** TTT **A** TTTGGCGGTGGAGGCGG
dATP-DT1 template	ACGCACGCACAAGCCACGGACCAAA **T** AAAGGCGGTGGAGGCGG
dCTP-DT1 template	CCACTCACTCTTACCTCAATCCTTT **G** TTTGGCGGTGGAGGCGG
dCTP-DT2 template	CCACTCACTCTTACCTCAATCCTTT **G** TTT **G** TTTGGCGGTGGAGGCGG
dGTP-DT1 template	GGAGTGAGTGTGAGGTGAATGGTTT **C** TTTGGCGGTGGAGGCGG
dGTP-DT2 template	GGAGTGAGTGTGAGGTGAATGGTTT **C** TTT **C** TTTGGCGGTGGAGGCGG

### Denaturing urea-PAGE gel

To demonstrate the exonuclease activity of the Taq polymerase on the template-probe complex, we used the dTTP assay under the same reaction conditions as described above (10 pmol dTTP-dT1 template, 10 pmol NDP1 primer in 25 μl reaction). We used denaturing 14% polyacrylamide-urea gel (7.5 M urea) to separate oligonucleotides at one base pair precision (23,32). As the probe did not penetrate into the gel in its original form containing the FAM fluorophore and the ZEN and iBFQ quenchers, we used 10 pmol dTTP-probe of identical sequence (5′ AGGACCGAGGCAAGAGCGAGCGA 3′) without these chemical modifications. We incubated the reaction mixtures at 95°C for 15 minutes followed by incubation at 60°C for 0–90 min. Reactions were terminated by adding 25 μl of the 2× formamide gel loading buffer (95% formamide, 0.025 w/v% Bromophenol Blue, 5 mM EDTA). The resulted 50 μl samples were heat denatured for 5 minutes at 95°C. The gel was prerun at 150 V at 45°C in TBE buffer for 1 h. Then 20 μl of each sample were loaded and the run was carried out at 100 V at 45°C in TBE buffer for 1 h 15 min. The gel was stained using 3333x GelRed in 100 mM NaCl solution.

### Kinetic analysis

When applying a kinetic analysis approach for dNTP quantitation, fluorescence data was plotted against time (s) and fitted with single or double exponential decay functions, as it is usual in enzyme kinetic analysis (Equations 1 and 2, respectively):

(1) $$\begin{equation*}F = A{\rm{*}}{e^{ - {k_{obs}}{\rm{ }}x}} + {F_0}\end{equation*}$$

(2) $$\begin{equation*}F = {A_1}{\rm{ *}}{e^{ - k{1_{obs}}{\rm{ }}x}} + {A_2}{\rm{*}}{e^{ - k{2_{obs}}{\rm{ }}x}} + {F_0}\end{equation*}$$

where *F* is the observed fluorescence, *x* is the variable (time), *A* and *A*_1–2_ are the amplitudes, *k*_obs_ and *k*_1–2obs_ are the rate constants of the observable fluorescence phases, while *F*_0_ is the *y* offset. Note, that the polymerase based dNTP quantitation method is not a PCR reaction, only primer elongation occurs without template amplification. Therefore, equations used in qPCR analysis are not applicable here. Also, note that as the fitted equations are decay functions, and we have an increasing signal, the resulting amplitude parameters are negative values. For a more straightforward interpretation, in further analyses we used the additive inverse of the fitted amplitude parameters, and simply referred to them as *A*, *A*_1_ and *A*_2_.

The resulting values (*A*_1–2_ and *k*_1–2obs_) were plotted against the corresponding dNTP concentrations to extract mechanistic information. Amplitude data were then fitted with the quadratic binding equation (Equation 3) describing 1:1 stoichiometry assuming no cooperativity:

(3) $$\begin{eqnarray*} {\rm{F}} &=& {F_0} + {{\rm{A}}_{quad}}\frac{{\left( {{E_T} + {L_T} + {K_d}} \right) \pm \sqrt {{{\left( {{E_T} + {L_T} + {K_d}} \right)}^2} - 4{\rm{\ }} \times {E_T} \times {L_T}} }}{{2 \times \ {E_T}}} \end{eqnarray*}$$

where *F* is the fluorescence or relative fluorescence of the sample, *F*_0_ is the intercept, *A*_quad_ is the total change in the amplitudes in the investigated specific dNTP concentration range, *E*_T_ is considered as the concentration of the polymerase:template:primer:probe complex, *K*_d_ is the apparent equilibrium constant of the specific dNTP dependent fluorophore release, *L*_T_ is the independent variable and it stands for the total added specific dNTP concentration.

### dNTP quantification based on kinetic analysis

Calibration curves for each specific dNTP were set up in the assay range and assay concentrations optimized for that specific dNTP. Raw fluorescence data was plotted against time and then fitted with Equation (2). As the blank reaction is better fitted by Equations (1) than (2) according to the Akaike information criterion (AIC), it was omitted from the further analysis. AIC is a statistical tool that, by estimating the amount of information lost by a model, allows us to estimate the risk of overfitting (the increasing number of parameters, the better the fit) and the risk of underfitting. The AIC formula for independent normally distributed random variables is described in Equation (4) (33,34),

(4) $$\begin{equation*} AIC = \left\{ {\begin{array}{l{@}{\quad}l} 2k + N*\ln \left( {RSS} \right), & {\rm when} \frac{N}{k} \ge 40\\ 2k + N*\ln \left( {RSS} \right) + \frac{{2k\left( {k + 1} \right)}}{{N - k - 1}}, & {\rm when} \frac{N}{k} < 40 \end{array}} \right. \end{equation*}$$

where AIC is the indicator upon the AIC calculation, *k* is the number of parameters of the fit (for single exponential fitting: *k* = 3, for double exponential fitting: *k* = 5), *N* is the number of data points within the curves, RSS is the residual sum of squares. Those low concentration points that could not be properly fitted with the double exponential function due to a small amplitude of the specific phase were also omitted and considered as data points under the detection limit. To obtain a calibration curve, the *A*_1_ values from the double exponential fits were plotted against the specific dNTP concentration. The appropriate range was fitted with linear function and then the parameters of the fitted linear function were used to quantify dNTP in biological samples. Results were normalized to 10^8^ CFU in case of bacterial or to 10^6^ cells in case of human samples.

### Construction of the nucleoTIDY software

We created a Python-based software termed ‘nucleoTIDY’, by using the Matplotlib, NumPy, SciPy, xlrd and xlsxwriter free packages without any modification. nucleoTIDY fits single and double exponential equations modified to result in positive amplitude values (Equations 5 and 6, respectively) using non-linear least squares minimization method built in the SciPy package.

(5) $$\begin{equation*}F = - A{\rm{*}}{e^{ - {k_{obs}}{\rm{ }}x}} + {F_0}\end{equation*}$$

(6) $$\begin{equation*}F = - {A_1}{\rm{*}}{e^{ - k{1_{obs}}{\rm{ }}x}} - {A_2}{\rm{*}}{e^{ - k{2_{obs}}{\rm{ }}x}} + {F_{0}}\end{equation*}$$

where the parameters are the same as in Equations (1) and (2). Further details are described in the Results section.

### Analysis of assay performance

Intra- and interassay coefficient of variation (CV), recovery and accuracy parameters were calculated as in Wilson *et al.* (24). Limit of detection (LOD) was calculated using two different methods. Either the lowest dNTP amount at which the progress curve could be fitted was considered as the detection limit. Or LOD = calibration line offset + 3*SD A1_low calibration points_, where the offset of the calibration line and the standard deviation of the A1 values for parallel measurements were considered at low dNTP concentration. These two methods usually gave similar values. Limit of quantitation was calculated similarly, except that 5*SD was taken into consideration instead of 3*SD.

### Assay validation using the tritium based polymerase assay

dNTP quantification using the tritium based polymerase assay was done as described previously (21,22,26) with a few modifications. Briefly, the reaction mixture (50 μl) contained 0.5 unit of exonuclease negative Klenow-fragment (Thermo Fischer Scientific) or 1 unit of recombinant Taq polymerase (Thermo Fischer Scientific), Klenow buffer or Taq buffer, 0.25 μM dNTP specific template, 0.25 μM primer, 2.5 μM [3H] dATP/ or [3H] dTTP (3 Ci/mmol) (American Radiolabeled Chemicals, Inc.) and 8 μl dNTP-extract or premixed dNTP for calibration. The calibration curve was prepared using 0, 0.25, 0.5, 1, 2, 4, 8 and 16 pmol dNTP/reaction. Incubation was carried out for 60 min at 37°C, or in case of Taq polymerase at 48°C. Next, the 50 μl reaction mix was spotted onto Whatman Grade 3MM Chr (Sigma) chromatographic disks (*d* = 24 mm). Disks were dried, washed with 5% Na_2_HPO_4_ for 3 × 10 min, rinsed once with distilled water and once again with 95% ethanol and then dried. Alternatively, 40 μl samples were spotted onto a prewetted positively charged nylon membrane (Zeta-Probe^®^ Membrane, Bio-Rad) using a vacuum-driven microfiltration apparatus (Bio-Dot, Bio-Rad). After 10 min of air-drying, immobilization of DNA was performed by applying 30 h of incubation at 80°C. The membrane was then washed similarly as described for the paper disks, dried, and cut into equal pieces.

The radioactivity retained by the disks/membrane pieces was measured in a liquid scintillation counter (Beckman). For dCTP and dGTP measurements, recombinant Taq polymerase (Thermo Fisher Scientific) was used, as the Klenow polymerase readily incorporates CTP and GTP from nucleotide extracts thus resulting in the overestimation of dCTP and dGTP concentrations (22).

### dUTPase treatment

For the determination of dUTP concentration, half of the sample was treated with dUTPase, then the difference between the dUTPase treated and nontreated parallel samples was considered. For dTTP measurement by the fluorescence method, dUTPase treatment was performed as part of the assay, as suggested by Wilson *et al.* (24) using 10–20 ng recombinant human (hDUT), or *Mycobacterium tuberculosis* dUTPase (mtDUT) per reaction. For the tritium method, half of the sample was treated with 10–20 ng recombinant mtDUT at 37°C for 1 h. dUTPase was inactivated by incubation at 95°C for 5 min. Recombinant hDUT and mtDUT were expressed and purified as described earlier in refs (35) and (36), respectively.

### Quantitation of dNTP from previously published bar graph

Data from Machon *et al.* (37) were quantified using the Web plot digitizer program (https://automeris.io/WebPlotDigitizer). dNTP values were calculated to 10^6^ cells based on the assay description in the article (200 000 cells/10 μl, 10 μl injected).

## RESULTS

### The polymerase based fluorescence dNTP quantitation assay by Wilson *et al.* fails in certain biological samples

We could well reproduce the assay developed by Wilson *et al.* (24) in water and with all components controlled. The performance including limit of detection, limit of quantification and accuracy we obtained was similar to the one reported in (24). However, when trying to determine dNTP levels in human (Figure 1) or bacterial (Supplementary Figure S1) cell lysates, the analysis failed as we obtained negative values. As this is far more the most attainable method to measure dNTP concentrations in a standard molecular biology laboratory, we set out to explore the background of this phenomenon and modify the assay so that it can be used in any biological samples.

The assay developed by Wilson *et al.* (24) is based on the TaqMan PCR principles. It utilizes a synthetic oligonucleotide template, a single primer and a dual-quenched fluorophore-labeled probe (Figure 1A). The fluorescent signal is generated by the cleavage of the 6-FAM fluorophore off the probe by the 5′-3′ exonuclease activity of the Taq polymerase. The concentration of limiting dNTP is expected to be directly proportional to the fluorescent signal. The calculation method they used assumed that the fluorescence intensity at a given time point should be used to calculate the limiting dNTP concentration in the sample using a linear calibration curve measured in water. We observed, however, that the reaction curves containing biological samples consistently run below the calibration curves obtained in water. Even if the standard dNTP solutions used for calibration were added directly into the biological samples, the same problem arose (Figure 1B). Besides, the kinetics of the reaction curves containing biological samples seemed to be slower compared to the calibration curves measured in water. The latter was also noted by Wilson *et al.* (24) and therefore, they suggested that the fluorescence results should be read out upon the completion of the reaction, approximately at 15 min for dGTP, dCTP, dTTP and at 20 min for dATP. In our repeated experiments, the reaction was not completed within 20 minutes neither for dGTP (Figure 1B), nor for other nucleotides.

We also observed that the reaction curve representing the zero calibration point (i.e. sample not containing the limiting dNTP species, ‘blank’) appeared kinetically faster than the curves representing the lower concentration calibration range. As a result, the blank curve exhibited higher fluorescence value at 20 min than some of the calibration points (Figure 1B). Upon extraction of the blank reaction suggested by the authors of ref. (24), the reaction curves reached a maximum followed by a fluorescence decrease (Figure 1C). Reading out the fluorescence at 15 min, we obtained the calibration curves presented in Figure 1D.

In conclusion, the suggested calibration method and single point read out cannot be used to obtain quantitative results for dNTP concentrations in biological samples.

#### Kinetic analysis

Figure 1B and C suggested more than one kinetically separable ongoing processes in the reaction mixture. Indeed, longer records unequivocally reveal that a substantial slow process is present even if the limiting dNTP concentration to be measured is zero (Figure 1E). We observed this phenomenon for all dNTPs in most samples including human (Figure 1) and bacterial ones (Supplementary Figure S1). We applied two different hot-start Taq polymerases with similar results: AmpliTaq Gold™ (Thermo) used by Wilson *et al.* (24) (Figure 1), and VWR^®^ TEMPase Hot Start DNA Polymerase (Supplementary Results andSupplementary Figure S1). The observed phenomenon did not depend on either the used template (dT1 template for 0–10 pmol dNTP and dT2 template for 0–20 pmol dNTP in Figure 1 and Supplementary Figure S1, respectively) or the used qPCR instrument (we used three different ones from Bio-Rad, Thermo Fisher Scientific and Agilent).

### Background phenomenon originates from the polymerization independent 5′-3′ exonuclease activity of Taq polymerase

To reveal the origin of the substantial slow process in the DNA elongation curve, we eliminated the assay components one by one (Figure 2). Figure 2A shows the resulting reaction curves while Figure 2B schematically indicates the macromolecular complexes formed in each set of conditions. A large fluorescence signal change occurs in all samples that contain polymerase, template and probe together (Figure 2). This indicates that the observed signal change is the result of a polymerase catalyzed reaction on the template-probe complex (TP complex), practically a polymerization independent 5′-3′ exonuclease activity. The presence of the primer seems to accelerate this reaction (cf. curve 2 versus curve 4 in Figure 2A). We came to the same conclusion using the VWR^®^ TEMPase Hot Start DNA Polymerase (Supplementary Figure S2). To confirm the polymerization independent 5′-3′ exonuclease activity of the Taq polymerase on the TP complex, we analysed the different reaction mixtures using denaturing 14% polyacrylamide-urea gel electrophoresis (Figure 2C). We set up reaction mixtures without dNTPs added (equal to condition 3 in Figure 2B) and run a time course (0, 20, 50 and 90 min). Primer (14 bp), template (43 bp) and probe (23 bp) were loaded separately to make the bands in the lanes containing all these three components identifiable. At *t* = 0 min, the primer, the template and the probe run at the height of their respective controls, while at later time points, a band appears below the size of the probe (red arrow) indicating the exonuclease activity of the Taq polymerase. We also applied a reaction condition where only the limiting nucleotide, dTTP was missing from the reaction mixture while all non-limiting dNTPs were added (condition 2 in Figure 2B). In this case, the elongated primer (yellow arrow) indicates the incorporation of the non-limiting dNTPs while the hydrolyzed probe also appears below the intact one. As a control, we performed a reaction with all assay components included (condition 1 in Figure 2B). In this case, the primer is fully elongated generating the complementary strand of the template DNA (green arrow). Note, that the running temperature of the urea gel (45°C) did not allow the complete denaturation of the template-length double stranded DNA (*T*_m_ = 80–22.5 = 57.5°C in the applied urea concentration (32)) resulting in a wide band. The polymerization independent 5′-3′ exonuclease activity of Taq polymerase was previously reported on substrates consisting of a template and a 5′ hybridized oligonucleotide (38). It was also reported that the presence of a second oligonucleotide annealed upstream to the first oligonucleotide enhances this exonuclease activity 3-fold, even if dNTP-s are not present (38), probably by promoting a more productive conformation of the polymerase-substrate complex. Our fluorescence observations in Figure 2A agree with the previously reported properties of the polymerization independent 5′-3′ exonuclease activity of Taq polymerase. Also supported by the observation of the time dependent hydrolysis of the probe in the gel electrophoresis experiment we identify the polymerization independent 5′-3′ exonuclease activity of Taq polymerase as the source of the slow process in the full reaction curve.

### Kinetic analysis of the assay curves enables the separation of the polymerization dependent and independent processes

The fluorescence time curves obtained in the assay describe the elongation of one primer per enzyme. This is ensured by the single thermal cycle setup and also by the slight excess of enzyme over the template, primer and probe added in equal quantities. The dNTP to be measured is substoichiometric compared to the enzyme-template-primer-probe complex (ETPP) in the 0–10 pmol range and superstoichiometric above. The treatment of the obtained progress curves with exponential equations serves as a tool to separate the polymerization—and dNTP—dependent process from the background so that a measure directly proportional to the concentration of the limiting dNTP species could be extracted. The blank reaction and all the other reactions arising from incomplete reaction mixtures shown in Figure 2 could be fitted with single exponential function (Equation 1). The curves obtained at various concentrations of the limiting dNTP could be well described with double exponential fits (Equation 2). We performed the AIC analysis, which indicated that using the double exponential model is justified over the single exponential one for reaction curves arising from the incorporation of any of the dNTP species. The observed rate constant (*k*_obs_) of the blank reaction approximated the *k*_obs_ of the second, slower phase of the specific dNTP containing reaction. Fittings revealed that the reactions go into real completion only around 1000 min by using the AmpliTaq Gold™ polymerase (Figure 1E) and around 400 min by using the VWR^®^ TEMPase Hot Start DNA Polymerase.

We investigated the kinetic parameters of the reaction curves in a broad dNTP concentration range (0–25 pmol specific dNTP/reaction which equals to 0–1 μM specific dNTP) for dTTP using the dT1 template (Figure 3). As shown in Figure 3A, the concentration dependence of the obtained *k*_obs_ values indicates two kinetically well separated processes. The *k*_obs_ of the fast phase (*k*_1obs_) exhibits strong dTTP concentration dependence (Figure 3A) indicating a dNTP incorporation dependent fluorescence signal change. The concentration dependence of *k*_1obs_ differs in the substoichiometric and superstoichiometric dNTP ranges (stoichiometric ratio is reached at 10 pmol/0.4 μM dTTP)) (Figure 3A). Below 10 pmol/0.4 μM dTTP, the observed rate constants exhibit a hyperbolic concentration dependence followed by a linear concentration dependence at superstoichiometric dTTP concentrations. The *k*_obs_ values of the slow phase (*k*_2obs_) are in the same order of magnitude as the single *k*_obs_ of the blank reaction (Figure 3A). A closer look at the concentration dependence of *k*_2obs_ (Figure 3B) reveals that the *k*_2obs_ varies between the polymerization independent hydrolysis values exhibited in the presence and in the absence of primer (cf. complexes 3 and 4 in Figure 2B).

The concentration dependence of the raw amplitudes, and the relativized amplitudes of the two phases are shown in Figure 3C and D, respectively. As expected, the amplitude of the polymerization dependent fast phase increases, while the amplitude of the slow phase decreases with increasing dNTP concentration (Figure 3C and D). The polymerization dependent relA1 goes into saturation at 7 pmol dTTP (see quadratic fit analysis in the figure legend of Figure 3D) and reaches a maximum of 0.7 (Figure 3D). 10 pmol template-probe-primer complex is present in the assay and thus, the theoretical maximum of limiting dNTP to be incorporated using the dT1 template is 10 pmol. The saturation of the specific incorporation reaction at 7 pmol dTTP indicates that 30% of this complex is not available for specific dNTP incorporation. This portion of the fluorescence signal is generated in the competing polymerization independent reaction.

Another example of the kinetic characterization of this assay is shown in Supplementary Figure S3 using dCTP as the dNTP to be quantified, a longer template (dT2) and the faster VWR® TEMPase Hot Start DNA Polymerase. Similar conclusions can be drawn from this set of experiments. The relative amplitudes indicate that 20% of the signal is generated in the polymerization independent process (Supplementary Figure S3).

### The analysis of the fast phase amplitudes yields reliable readout

**Scheme 1.**
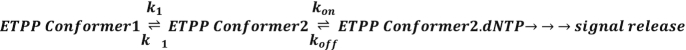
Conformational selection mechanism for dNTP binding to the enzyme-template-primer-probe complex (ETPP).

By investigating the kinetics of the assay curves for all dNTPs, we obtained different behavior for dCTP and dGTP incorporation into dT1 templates than for dTTP, dUTP and dATP incorporation using the AmpliTaq Gold™ polymerase (Figure 4A). The *k*_1obs_ hyperbolically decreases with increasing dCTP or dGTP concentration up to reaching stoichiometry with the template. On the other hand, the *k*_1obs_ hyperbolically increases with the increasing dNTP concentration for the three other dNTPs (Figures 4A and 3A). In superstoichiometric dNTP concentrations, the *k*_1obs_ of all dNTPs shows linear increase with increasing dNTP concentrations (Figures 4A and 3A). This phenomenon likely indicates conformational selection mechanism (39) for dNTP binding to the enzyme-template-primer-probe complex (ETPP) (Scheme 1). In this case, there exist an equilibrium between two conformers of the ETPP complex only one being competent for dNTP binding. The substoichiometric concentration dependence of *k*_1obs_ will then depend on the ratio of the constant *k*_1_ and *k*_off_ for each dNTP (Scheme 1): it will either be a hyperbola with an increasing (*k*_1_ > *k*_off_) or a decreasing (k_1_ < k_off_) slope (for more detailed explanation of conformational selection check ref. (39)). This would explain the different behavior of *k*_1obs_ in the substoichiometric concentration range of dCTP/dGTP and dTTP/dUTP/dATP. The underlying mechanism of the phenomenon we observe can be explained by the discrete state model of DNA polymerase reaction created on the basis of kinetics data and crystal structures of polymerase–DNA and ternary complexes (40). Based on structural data, two different conformers of the polymerase–DNA complex (equivalent of ETPP here) are distinguished (40). According to the model, there exists an equilibrium between a stacked and an unstacked state of the template base in the polymerase–DNA complex (40). In the stacked conformer, the template base is organized so that it base pairs with the incoming nucleotide while in the unstacked conformer, the incoming nucleotide cannot base pair with the template (40). The incoming dNTP will therefore preferentially bind to the stacked state on the end of the primer strand as if it were already part of a double-stranded DNA. The base pairing and stacking of the incoming dNTP with the complementer nucleotide is a driving force in the polymerase reaction and serves fidelity. We suggest that the conformational selection phenomenon we observe in our kinetics data occurs at the level of this pre-existing equilibrium between the stacked and unstacked template base. The fact that the incorporation kinetics of the nucleotides falls into two categories according to the number of H-bonds present between base pairs (dCTP/dGTP versus dTTP/dUTP/dATP) supports this suggestion. A pre steady-state kinetics study that uses Förster resonance energy transfer (FRET) to investigate the conformational dynamics of Taq DNA polymerase during nucleotide binding and incorporation could also distinguish a rapid conformational equilibrium in the polymerase-DNA complex prior to dNTP binding (11–36 s^−1^) (41). The authors presumed that only one of these conformations is competent for dNTP binding (41). Moreover, the study showed that this equilibrium remained unchanged using a non-extendable DNA template (41). These experimental findings are in line with our observations.

Figure 4B shows that the dNTP concentration dependence of *k*_2obs_ is not sensitive to the dNTP species.

Figure 3D indicates that the specific, dNTP incorporation dependent signal release competes with the aspecific, dNTP independent signal release. As the concentration of the specific dNTP increases in the reaction mixture, the amplitude contribution of the specific reaction increases, while that of the aspecific reaction decreases. Figure 4C and D shows that the relative amplitudes and amplitudes of the fast reaction, respectively, exhibit strong and consistent dNTP concentration dependence for all five dNTPs measured. The linear phase of the amplitude curve (Figure 4D) can be used as a reliable readout for dNTP quantification. By using the VWR^®^ TEMPase Hot Start DNA Polymerase, the same conclusions can be drawn for k_1obs_, k_2obs_ and the A_1_ amplitude appropriate for dNTP quantification (Supplementary Figure S4). Due to the differences in the kinetic mechanism of incorporating various dNTP species by DNA polymerase, the assay conditions should be modified to arrive to the largest possible dynamic range in amplitude based dNTP quantitation.

### Novel analysis method and altered assay conditions eliminate disturbing effects

The dNTP concentration dependent competition of the dNTP dependent and independent reactions, and the resulting observed rate and amplitude changes explain the disturbing effects in the original method presented in Figure 1. As the amplitude of the dNTP independent reaction is largest in the blank reaction (cf. Figures 1E or 3C) and at low dNTP concentration, the subtraction of the blank reaction at the apparent saturation point (fluorescence read out at 15 min) results in a negative value (cf. the 0.5 pmol, and the 0.75 pmol calibration point in Figure 1D). The sample matrix may cause similar effect by inhibiting the dNTP-independent kinetics more than the dNTP dependent kinetics (latter also observed by Wilson *et al.* (24)). During our hundreds of trials, we observed that the incorporation rates of the various dNTPs are different and are also greatly affected by the biological matrix. The amplitudes of the polymerization dependent fast phase, however, are reliable and yield high quality calibration curves for dNTP quantitation. Figure 4D shows the result of the kinetic analysis of the same data set as the one shown in Figure 1.

However, as it is observable in Figure 4C and D, few concentration points define the linear range which can be used for the amplitude based analysis. To broaden the dynamic range for amplitude based dNTP quantitation, we varied the template, probe, and NDP1 primer concentration between 10–20 pmol / reaction, the MgCl_2_ final concentration between 2.5 and 5 mM, and the AmpliTaq Gold™ polymerase concentration between 0.413 and 0.825 units/reaction.

For optimizing data acquisition, different cycle lengths and cycle numbers were applied in the qPCR instrument. ‘Split time’ data acquisition was also applied: the first half of the cycles was set up with a short cycle length for the optimal detection of the fast reaction phase, while the later cycles were longer for the optimized detection of the slow phase.

We observed that an increase in the primer-template-probe concentration in the reaction broadened the linear range of the calibration curves, while the detection limit of the assay did not change. By optimizing the cycle length, and by introducing split time data acquisition, the detection limit also improved as the quality of the fitting improved. This change in settings prevented overfitting of the slow phase.

In our experiments, we mostly used oligonucleotides ordered with standard desalting purification. We observed that the applicable calibration range varied from batch to batch. This is probably due to the fact that shorter oligonucleotide variants may also be present in the batches. To avoid this variation, we suggest the use of PAGE purified oligonucleotides, as it was also done by Wilson *et al.* (24) However, if cost reduction is a goal, the applicable calibration range should be determined for each batch. Also note that increasing the primer-template-probe concentration (cf. Table 1 dTTP data) will broaden the linear range of the measurement. This proved to be especially important for dATP measurements, where the aspecific reaction can more efficiently compete with the relatively slow dATP incorporation reaction.

In case of dCTP and dGTP, which are incorporated faster by both DNA polymerases than the other dNTPs, the decrease of polymerase concentration and of the assay temperature improved the detection limit as the reaction became slower (cf. Table 1, dGTP data). For dTTP, decreasing the assay temperature improved the detection limit too (cf. Table 1 dTTP data). In contrast, the original, relatively high concentration of polymerase needed to be retained in dTTP measurements. For dATP, decreasing either the polymerase concentration or the assay temperature was disadvantageous, as they decreased the already low rate of dATP incorporation.

We found that an increased MgCl_2_ concentration counteracted part of the kinetic inhibition always observed in biological samples. Mg^2+^ chelation may result from high pyrophosphate and/or nucleotide concentration in cell lysates. The addition of extra MgCl_2_ was absolutely necessary for dATP quantitation in samples with low dATP concentrations. In case of dATP, data acquisition had to be much longer even with the addition of extra MgCl_2_ than for the other nucleotides (cf. Table 1).

In some samples, we also observed a lag phase (100–200 s long) which could be decreased by adding extra MgCl_2_. Another solution to overcome this disturbing effect is to dilute the sample where it is possible.

In the radioisotope incorporation assay, a large overestimation of dNTP levels was observed in the background of high concentrations of rNTP (22). Under the optimized measurement conditions and using the kinetic data analysis, our method quantifies dNTP reliably even in a large excess of rNTP. dCTP, dTTP, dATP and dGTP recovery in the presence of 1000× excess of CTP, UTP, ATP and GTP was 105.7 ± 4.9%, 95.3 ± 0.7%, 101.3 ± 15.4% and 80.4 ± 0.1%, respectively. The recovery of dGTP in the presence of 500× excess of GTP was 100.3 ± 1.3%. We offer optimized conditions in Table 1 and Supplementary Table SI for AmpliTaq Gold™ and VWR^®^ TEMPase Hot Start DNA polymerases, respectively. Statistical parameters of the improved assay can also be found in Table 1. The detection limits for individual dNTPs are similar to those reported by Wilson *et al.* In average, the recoveries are also similar. However, we observed that the recovery depends on the concentration and on the particular sample (cf. Table 1). Therefore, it is useful to include a standard addition control when measuring a yet untested type of sample.

#### dUTP concentration determination

Wilson *et al.* suggested to digest the dUTP content of the sample and calculate dUTP concentration as the difference between the non-treated (dTTP + dUTP) and the dUTPase treated (dTTP) measurements. They used human dUTPase which did not work for us in the biological sample. The dUTPase catalysed reaction is accelerated by Mg^2+^ and some dUTPases, including the human dUTPase, contain structural Mg^2+^ binding site(s), as well (42). To hydrolyze dUTP in biological samples, we suggest the use of a dUTPase that does not contain any structural Mg^2+^ binding site (e.g. *Mycobacterium tuberculosis* dUTPase (43)). Even though we could eliminate dUTP in the biological samples using dUTPase digestion, we still not gained reproducible dUTP concentrations. When we created various predetermined dUTP:dTTP ratios in the reaction mixtures, we failed to recover dUTP if the dTTP:dUTP ratio was >1.5. We suggest that the reason behind this phenomenon is the kinetic difference in dTTP and dUTP incorporation into DNA. Although not really emphasized in the literature, dUTP incorporation is significantly slower than dTTP incorporation under the same conditions (cf. Figure 4A). According to our experience, this makes the proper detection of dUTP unmanageable. For quantitative detection of dUTP, we suggest using the radioactive end point assays.

### Validation of the assay

**Figure 5.**
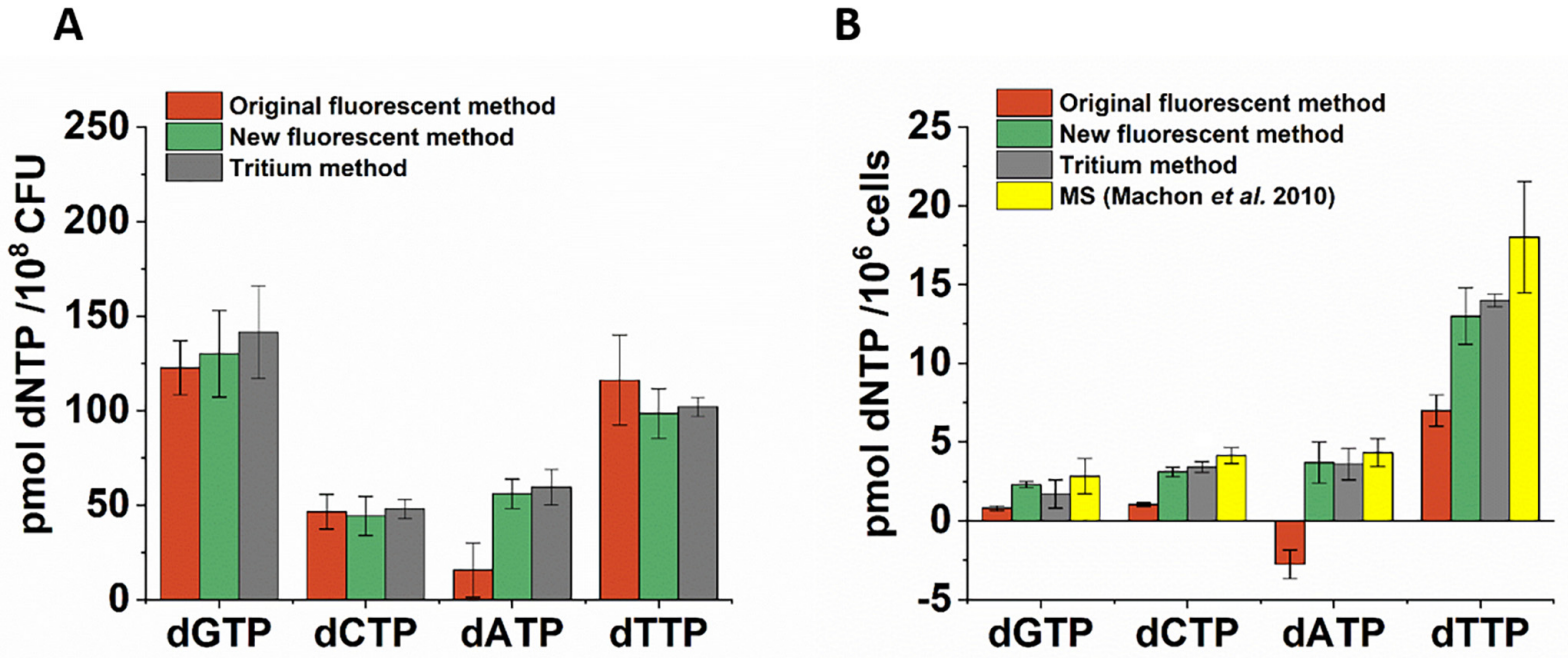
Comparison of the improved dNTP quantification method with the original, the radioactive isotope based and the MS based ones. (**A**) dNTP quantification in *Mycobacterium smegmatis* samples. Data represent mean and standard error of two biological parallels and 3 technical repeats each. The presented dNTP quantities were determined for the same sample using the three different methods. The same raw curves were quantified using the original and our new, improved fluorescent methods. (**B**) dNTP quantitation in human MES-SA samples. Data represent mean and standard error of 4 biological parallels and at least three technical repeats, for dCTP measured using the tritium method three technical repeats. For MS spectrometry, data were extracted from the results published in Machon *et al.* (37).

For comparison, we subjected the same biological samples to the following measurements: (i) the original Wilson *et al.*, (ii) the here improved fluorescence and iii) the well-established radioactive (21,22) one (Figure 5B). We used cell extracts from logarithmically growing *Mycobacterium smegmatis* culture. The improved fluorescence and the radioactive methods yielded indistinguishable dNTP concentrations for all four canonical dNTPs, while the original fluorescence assay detected dATP with much less certainty (Figure 5A). This bacterial sample contains particularly high concentrations of dNTPs and therefore, the disturbing effects in the original method are manifested only with the most problematic dNTP, dATP. However, when using human cell extracts which contain lower dNTP concentrations, the comparison yields large differences. We chose MES-SA cells as reliable literature data is available on the quantitation of dNTPs by MS for this cell line (37). Figure 5B shows the results of comparing our measurements analysed by i) the original Wilson *et al.* method; ii) the improved fluorescence method and iii) the tritium method with the MS data from (37). Importantly, at low dNTP concentrations, our method permits dNTP quantitation while the original Wilson *et al.* method does not. The result of our novel analysis corresponds well with the tritium and the MS data, considering that the MS experiments were done separately and that these are widely different approaches.

### nucleoTIDY, a tool for streamlined kinetic analysis of fluorescent dNTP incorporation curves

**Figure 6.**
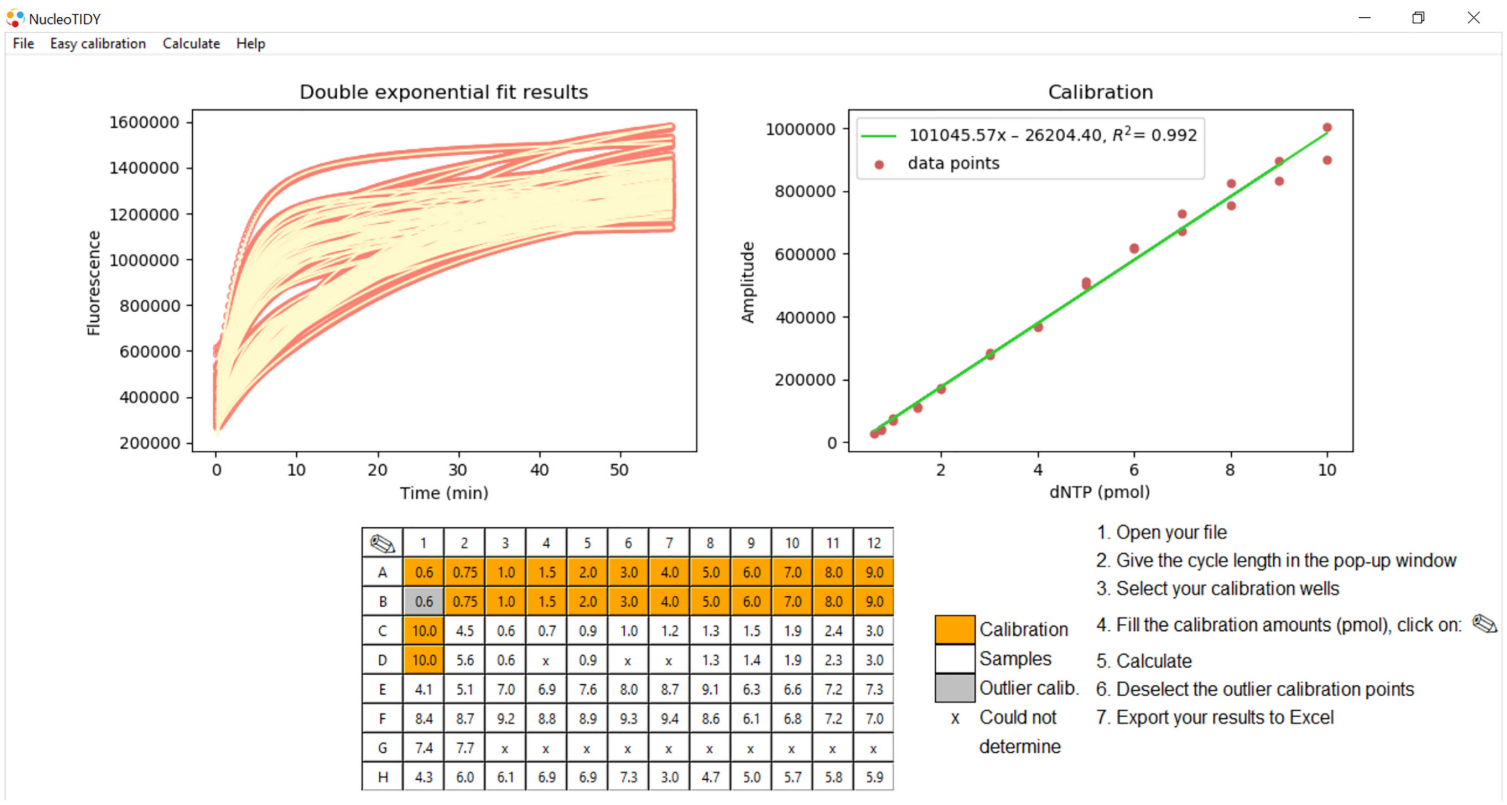
The graphical interface of the nucleoTIDY software displays instructions for use and the results of the kinetic analysis as well. The software fits the raw measurement curves (left panel, salmon data points) with double exponential equations (left panel, yellow lines). Once the user defines the calibration wells and amounts, hitting the Calculate button in the menu bar results in linear regression of the calibration points (shown in the right panel) and the amount of dNTP in the default sample wells are calculated (shown in the 96-well panel). The user can refine the calibration by removing outlier data points (grey wells in the 96-well panel). In case the raw data are not appropriate for converting into results, an x is shown in the 96-well panel. The exported Excel file contains all the results and fitted parameters as well as information on calculation failure, if any. The output file also specifies if the result fell outside of the calibration range. This figure demonstrates a dCTP measurement using dT1 template and VWR^®^ TEMPase Hot Start DNA Polymerase at 55°C.

The method presented herein is the most accessible and most high-throughput of all existing dNTP quantitation methods. Still a major drawback in using it might be the necessity of kinetic analysis in which most potential users are inexperienced. To overcome this complication, we implemented our evaluation algorithm into the software nucleoTIDY, a Python-based stand-alone executable program able to read and kinetically analyze the exported qPCR runs. nucleoTIDY is a user-friendly software which provides a graphical user interface (Figure 6) displaying the 96-well assay plate as well as the running instructions. The software is made in a way, however, that even the inexperienced user can judge the quality of the kinetic analysis through the process. Several checkpoints were built in to prevent the further use of unreliable results when applying the software to analyse low quality raw data. The cause of failure of the analysis most often is the overfitting of a single exponential curve with double exponential function which results in two inseparable kinetic phases. To avoid that error, the nucleoTIDY software fits all curves with both single (Equation 5) and double exponential functions (Equation 6). The software then compares the single and double exponential fitting parameters using the Akaike information criterion (AIC) (Equation 4) and selects the appropriate fitting method.

If a single exponential fit is more appropriate than a double exponential fit, i.e. AIC (single exponential fit) < AIC (double exponential fit), then those curves will be excluded from further analysis, and nucleoTIDY displays an ‘x’ in these wells. Following the fitting process, nucleoTIDY sorts out and compiles the fitted parameters. dNTP amounts are calculated based on the *A*_1_ parameter as discussed earlier. The user defines the wells that contain the calibration points (represented as orange wells in Figure 6). Then by hitting the calculate button, the calibration points are calculated, plotted and fitted with a linear equation. The software displays the calibration chart so that the user can evaluate its quality. There is a possibility to refine the calibration curve via excluding outlier data points (grey wells in Figure 6). The calculated result, i.e. the amount of dNTP in each well, is displayed in the graphical representation of the 96-well plate.

The in-built analysis checkpoints include the recognition of (i) inseparable kinetic phases discussed above; (ii) a high error (error > value) in the key parameter *A*_1_; (iii) *k*_1obs_ being within the error range of the average of *k*_2obs_ values of the entire plate; (iv) *k*_2obs_ being lower, than 10^−5^ s^−1^; (v) *A*_1_ being negative (inverse run of the exponential) and (vi) a small signal change, i.e. the total fluorescence signal change of the curve is 50% smaller than the total fluorescent signal change of the lowest calibration point. If the analysis fails at these checkpoints, nucleoTIDY displays an ‘x’ in the respective wells to prevent further use of low quality data. If the raw curves contain a lag phase, data points of the lag phase are eliminated to improve the quality of fitting. The output of the process can be chosen by the user: results can be exported into Excel or saved in the software for later use. The xlsx report of the analysis contains the explanation of failure for each well and thus makes the kinetic analysis transparent. The software processes one 96-well plate in some seconds saving the user significant time and effort in data analysis. nucleoTIDY is freely available at http://nucleotidy.enzim.ttk.mta.hu/. A tutorial video presents its simple use at the same site.

## DISCUSSION

We developed an improved fluorescent dNTP quantification method which performs well in biological samples even at low dNTP concentrations. Although this method requires the rigorous kinetic analysis of the fluorescence time courses recorded by the qPCR instrument, the nucleoTIDY data analysis software we developed (http://nucleotidy.enzim.ttk.mta.hu/) makes the method user friendly and readily applicable in any molecular biology laboratory.

We identified and addressed three major drawbacks in the basically ingenious method published earlier by Wilson *et al.* (24) that hindered the production of reliable results. First of all, due to the slow, nonspecific cleavage of the fluorescent probe by the Taq polymerase, no endpoint of the dNTP incorporation reaction can be reliably detected. Secondly, the rate of incorporation of various nucleotides widely varies, dATP being the slowest and dCTP/dGTP being the fastest of dNTPs. Finally, other components in the biological sample slow down the reaction in an unpredictable and unreproducible way. We efficiently resolved these issues by taking the whole progress curve into consideration and by the optimization of the assay conditions for each dNTP. The kinetic treatment of the qPCR curves allowed us to distinguish the dNTP incorporation process from background reactions. This way, the amplitude of the separated dNTP incorporation process does correlate with the amount of available dNTP in the reaction mixture. The altered kinetic properties of dNTP incorporation in biological samples may originate from the competition of dNTPs with rNTPs, (d)NDPs and (d)NMPs for binding to the polymerase. Although this competition decreases the progress rate, the reaction amplitude is not affected. Another reason for a slower observed dNTP incorporation kinetics can be Mg^2+^ depletion by chelating molecules in the biological sample. Adding extra MgCl_2_ helped the otherwise extremely slow dATP measurement and increased sensitivity.

We applied two different hot start Taq polymerases throughout the study (AmpliTaq Gold™ and VWR^®^ TEMPase Hot Start DNA Polymerase). Both exhibited background exonuclease activity and were affected by the matrix effect of the biological sample. However, there was a relatively large difference in the incorporation and in the background exonuclease reaction kinetic rates between the two polymerases. AmpliTaq Gold™ proved to be slower than VWR® TEMPase Hot Start DNA Polymerase (both the specific and the background reactions). The faster VWR^®^ TEMPase Hot Start DNA Polymerase is time saving, more cost effective and provides the same sensitivity as AmpliTaq Gold™. Other DNA polymerases can also be used, however, the measurement conditions will probably require optimization.

The reliability of the quantitative detection of dUTP in this assay highly depended on the dUTP:dTTP ratio due to the different incorporation kinetics of these nucleotides. Several pieces of evidence in the literature reinforce that DNA polymerases distinguish dTTP and dUTP (44,45). Commercial PCR protocols also suggest using double concentration of dUTP when replacing dTTP in the dNTP mixture (e.g. the protocol for AmpliTaq Gold™ polymerase). The comparison of the non-treated (comprising dTTP + dUTP) and the dUTPase treated (comprising only dTTP) data points may be suitable for the indication of large amounts of dUTP in a sample. Nevertheless, we suggest that neither the original fluorescence assay nor the present method is applicable for dUTP quantification. Instead, we suggest using the tritium based assay (which is a real endpoint assay) or an MS method for dUTP quantitation. We also strongly suggest that any samples should be treated with dUTPase before dTTP quantitation.

dNTP recoveries depended on sample concentration and cell type (cf. Table 1). Therefore, we strongly suggest to include standard addition as a control, especially when measuring a yet untested type of sample. Adding a known amount of dNTP at the time of extraction is also useful to detect and quantitate possible degradation of dNTPs. Another method to ascertain sample stability is to compare first results to those obtained using HPLC or, HPLC coupled MS methods (e.g. those presented in (17,20,37)). From HPLC, it will be possible to judge the extraction efficiency (since NTPs vary much less than dNTPs in different cells) and if NTPs are degraded to NDPs.

We calculated that we measured 100–500-fold difference in dNTP concentration between mycobacterial and human cells considering that the cell volume of a typical bacterium is 1 μm^3^ and that of a typical human cell is 1000 μm^3^. When measuring high concentrations of dNTP, the performance of the original method approaches that of the new one. Except that the quantitation of dATP levels remains less reliable. However, the determination of dNTP concentrations from mammalian cells unquestionably requires our assay developments.

This is the first report on the dNTP pool balance of a mycobacterial species. Interestingly, dGTP is the most abundant of dNTPs in *Mycobacterium smegmatis* while in other species it is the least abundant one e.g. in *Escherichia coli* (46) or in yeast (47). This difference may be due to the fact that the GC/TA ratio in the genome of *Mycobacterium smegmatis* is especially high (67%).

We developed the nucleoTIDY software to make the presented assay improvements attainable for users. The software can handle the output files of qPCR instruments from three major worldwide suppliers. We invite users of other types of qPCR instruments to contact us in order to include the capability of handling their output files in the software, as well.

## DATA AVAILABILITY

The nucleoTIDY software is available for download at http://nucleotidy.enzim.ttk.mta.hu/.

## Supplementary Material

gkaa116_Supplemental_File
