# Supplementary material for: A user-friendly, high-throughput tool for the precise fluorescent quantification of deoxyribonucleoside triphosphates from biological samples
Source: Nucleic Acids Res. 2020 Feb 27;48(8):e45. doi: 10.1093/nar/gkaa116 (PMC7192609; doi:10.1093/nar/gkaa116)
Supplement: gkaa116_Supplemental_File [file gkaa116_supplemental_file.pdf]

Supplementary material to

A user-friendly, high-throughput tool for the precise fluorescent quantification of deoxyribonucleoside triphosphates from biological samples

Judit Eszter Szabó[1,2*], Éva Viola Surányi[1,2], Bence Sándor Mébold[1], Tamás Trombitás[1,2], Mihály Cserepes[1], Judit Tóth[1*]

[1] Institute of Enzymology, Research Centre for Natural Sciences, Budapest 1117, Hungary
[2] Department of Applied Biotechnology and Food Sciences, Budapest University of Technology and Economics, Budapest 1111, Hungary
* To whom correspondence should be addressed. Tel: +36 1 382 6793; Email: toth.judit@ttk.mta.hu Correspondence may also be addressed to szabo.judit.eszter@ttk.mta.hu.

The authors wish it to be known that, in their opinion, the first two authors should be regarded as joint First Authors.

Supplementary Figures

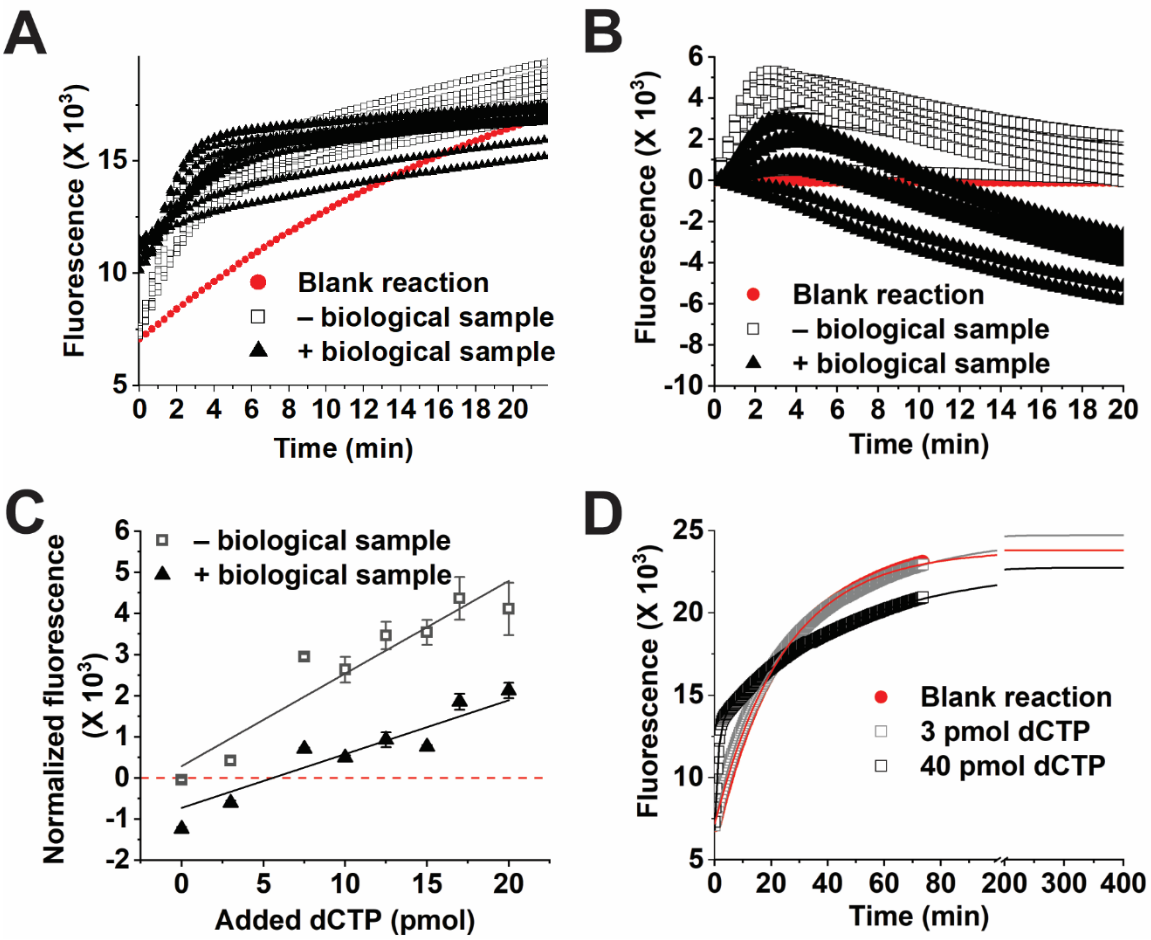

Figure S1 Measurement of cellular dCTP concentration in *Mycobacterium smegmatis* sample using the dCTP-dT2 template and TEMPase Hot Start DNA Polymerase at 55 °C Panel **A)** Raw reaction curves in the absence (hollow squares) and in the presence (black triangles) of *Mycobacterium smegmatis (M. smegmatis)* extract (1.5*10^8 cells / reaction). 0-40 pmol dCTP was added to the assays. Note, that the blank reaction (0 pmol dCTP) also produces signal which was corrected for by Wilson *et al.* via subtracting the blank curves from assay curves containing added dCTP or biological sample (shown in Panel B). **B)** Reaction curves corrected for according to Wilson *et al.* **C)** Calibration curve (0-40 pmol dCTP) in the absence of biological sample (hollow squares), and standard addition points in the presence of biological sample (black triangles) derived from reading the fluorescence in Panel C at 4 min. Continuous line is the linear fit to the calibration points yielding the following parameters: slope = 225 ± 32, intercept = 288 ± 396, R^2 = 0.87. Data and errors represent the average and standard deviation of technical parallels (n=2). **D)** Fitting the raw calibration curves (scatter plots) with exponential functions (continuous line). In case of the blank reaction (0 pmol dCTP), a single exponential equation (Eq. 1) could be fitted to the reaction curve, while in the presence of the specific dNTP (here, dCTP) the reaction could be well described with a double exponential equation (Eq. 2). The parameters yielded from the exponential fits are as follows: 0 pmol dCTP: A = -16609, k_{obs} = 6.7 *10^{-4} s^{-1}, y_0 = 23806; 3 pmol

dCTP: A_1 = - 5446, k_{1obs} = 1.5 * 10^{-3} s^{-1}, A_2 = -12021, k_{2obs} = 4.3 * 10^{-4} s^{-1}, y_0 = 24718; 40 pmol dCTP: A_1 = -6408, k_{1obs} = 1.5 * 10^{-2} s^{-1}, A_2 = -9513, k_{2obs} = 3.7 *10^{-4} s^{-1}, y_0 = 22748.

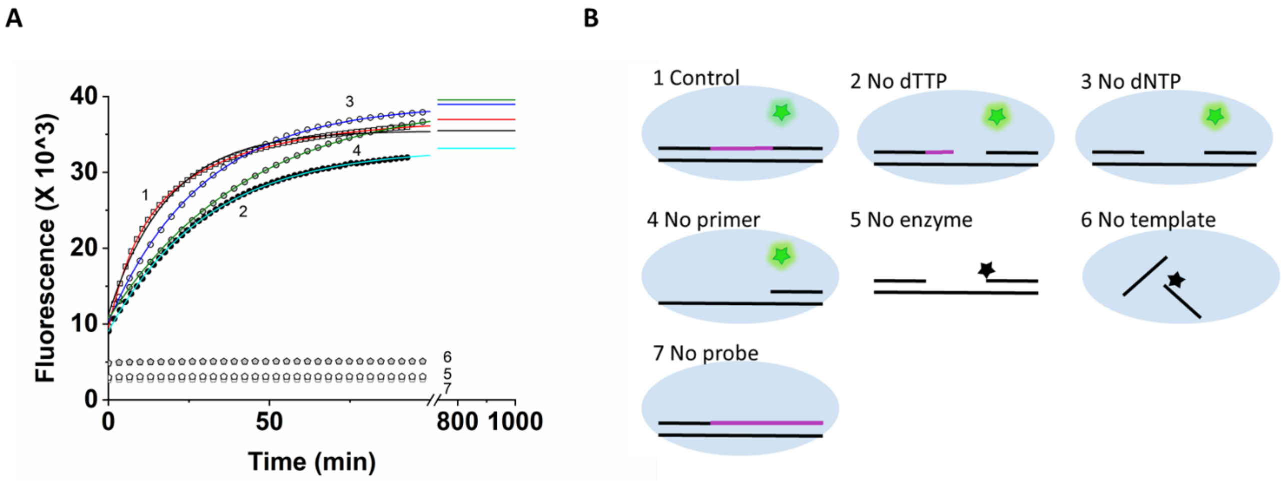

Figure S2 Assay background originates from dNTP incorporation independent 5'-3' exonuclease activity of TAQ polymerase A) To investigate the background of the assay using the TEMPase Hot Start DNA Polymerase, we chose the dTTP assay using the dT2 template. The data presented in Panel A represent the reaction curves obtained under the conditions schematically shown in Panel B. Continuous lines represent the exponential fits to the data. Parameters are as follows: 10 pmol dTTP (1), red : A_1 = -12508, k_{1obs} = 2.05*10^{-3} s^{-1}, A_2 = -15014, k_{2obs} = 4.85*10^{-4} s^{-1} y0 = 36952; 0 pmol dTTP (2), cyan: A_1 = -24118, k_{obs} = 5.45*10^{-4} s^{-1}, y0 = 33165; No aspecific dNTP added (3), blue: A_1 = -28676, k_{obs} = 5.71*10^{-4} s^{-1}, y0 = 38969; No primer added (4), green: A = -28902, k_{obs} = 3.88*10^{-4} s^{-1}, y0 = 39555. A single exponential function fitted to the "10 pmol dTTP" (1) data is shown as continuous black line. Note that the k_{obs} of the aspecific phase is about two times larger than that using AmpliTaq Gold polymerase. However, the observed rate constants relate to each other similarly as in the case of AmpliTaq Gold polymerase under the used conditions. **B)** Panel B schematically explains the constitution of the different assays. The incorporated dNTP-s are colored magenta.

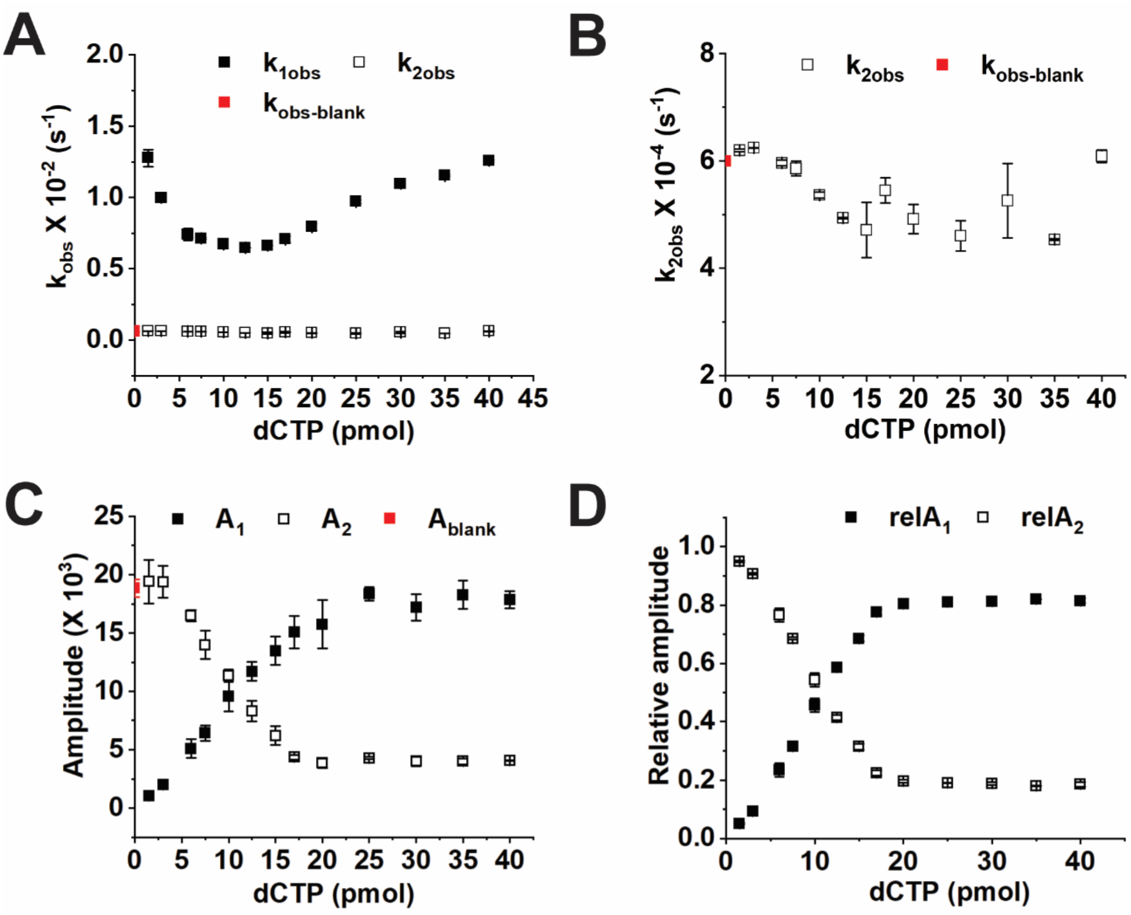

Figure S3 Background exonuclease activity of TEMPase Hot Start DNA Polymerase competes with specific dNTP incorporation in a dNTP concentration dependent manner using the dCTP-dT2 template at 55 °C A) Concentration dependence of the observed rate constants of the two kinetic phases in the assay (k_{1obs} and k_{2obs}, respectively). **B)** Concentration dependence of the observed rate constant of the slower phase (k_{2obs}). **C)** Concentration dependence of the amplitudes of the two phases (A1 and A2, respectively). Note, that the amplitude follows a hyperbola of which the initial linear phase can be used for calibration. **D)** Concentration dependence of the relative amplitudes of the two phases ($relA_1$ and $relA_2$, respectively). The amplitudes were normalized compared to the total amplitude of the reaction (A1+A2). Data and errors represent the average and standard error of the mean of technical parallels (n=2).

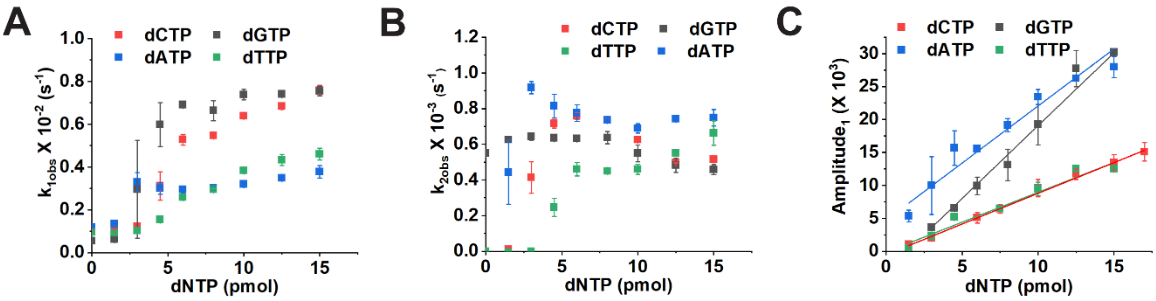

Figure S4 Separation of the reaction phases for precise quantification of dNTP-s using dCTP-dT2, dGTP-dT2, dATP-dT1 and dTTP-dT1 templates and TEMPase Hot Start DNA Polymerase A) Concentration dependence of the observed rate constants of the fast kinetic phases in the assay (k_{1obs}). **B)** Concentration dependence of the observed rate constant of the slower phase (k_{2obs}). **C)** Concentration dependence of the linear phase of the amplitudes of the fast, specific dNTP incorporation associated phase (A_1). Linear fits to the curves yielded the following parameters: dCTP, intercept = -530 ± 141, slope = 932 ± 36, R^2 = 0.99; dGTP, intercept = -3031 ± 127, slope = 2210 ± 32, R^2 = 0.999; dATP, intercept = 4719 ± 959, slope = 1733 ± 100, R^2 = 0.98; dTTP, intercept = -118 ± 734, slope = 906 ± 89, R^2 = 0.95. For dCTP, the same data points are presented in Figure 3. Data and errors represent the average and standard error of the mean of technical parallels (n=2).

Supplementary Table I.

	Template	Assay conditions	qPCR	Data acquisition	T (°C)	Calibration range	R^2	LOD[1] (pmol)	LOQ[2] (pmol)	Accuracy Low, High (%)	Interassay CV (%)	Intrassay CV (%)	Recovery		
													(%)	CFU or cell[3] /reaction	Sample type
**dTTP**	dT2	10 pmol TPP[4], 0.9 unit VWR® TEMPase 2 mM $MgCl_2$	Quant Studio 1	13 or 15 sec/cycle for 260 cycles	55	2-20 pmol	0.99	1.5	2.5	100 ± 2<br>99 ± 3	3.7 ± 2.5	3.1 ± 2.6	N.D	N.D	-
**dCTP**	dT2	10 pmol TPP[4], 0.9 unit VWR® TEMPase 2 mM $MgCl_2$	BioRad CFX96	13 or 17 or 19 sec/cycle for 260 cycles	55	1.5-20 pmol	0.99	0.7	1.2	109 ± 6<br>99 ± 1	4.3 ± 1.7	3.6 ± 2.0	102 ± 5	1 X 10^8	*Mycobacterium smegmatis*
**dGTP**	dT2	10 pmol TPP[4], 0.9 unit VWR® TEMPase 3.5 mM $MgCl_2$	BioRad CFX96	13 or 15 s for 260 cycles	55	3-20 pmol	0.999	1.4	2.4	103 ± 3<br>100 ± 1	6.1 ± 0.3	2.8 ± 1.2	110 ± 9	1 X 10^8	*Mycobacterium smegmatis*

[1]LOD calculated as follows: LOD= calibration line offset + 3*SD $A1_{\text{low calibration points}}$

[2]LOQ calculated as follows: LOQ= calibration line offset + 5*SD $A1_{\text{low calibration points}}$

[3]dNTP extract from the given number CFU (for bacteria) or cells (for eukaryotic cells)

[4]Template-primer-probe (TPP) complex
